# Non-linear regression models for time to flowering in wild chickpea combine genetic and climatic factors

**DOI:** 10.1186/s12870-019-1685-2

**Published:** 2019-03-19

**Authors:** Konstantin Kozlov, Anupam Singh, Jens Berger, Eric Bishop-von Wettberg, Abdullah Kahraman, Abdulkadir Aydogan, Douglas Cook, Sergey Nuzhdin, Maria Samsonova

**Affiliations:** 1 Peter the Great St. Petersburg Polytechnic University, 29 Polytechnicheskaya, St. Petersburg, 195251 Russia; 2 Program Molecular and Computation Biology, University of California, University Park, Los-Angeles, 24105 CA USA; 3 Commonwealth Scientific and Industrial Research Organization (CSIRO), Agriculture and Food, Underwood Ave, Perth, 6014 WA Australia; 4 Department of Plant and Soil Science, University of Vermont, 63 Carrigan Drive, Burlington, 05405 VT USA; 5 Deptartment of Plant Pathology, University of California, One Shields Ave, Davis, 95616-8680 CA USA; 6 Central Research Institute for Field Crops (CRIFC), P.O. Box 226, Ankara, 06042 Turkey; 7 Department of Field Crops, Faculty of Agriculture, Harran University, Osmanbey Campus, Sanliurfa, 63100 Turkey

**Keywords:** Wild chickpea, Model, Climatic factors, GWAS

## Abstract

**Background:**

Accurate prediction of crop flowering time is required for reaching maximal farm efficiency. Several models developed to accomplish this goal are based on deep knowledge of plant phenology, requiring large investment for every individual crop or new variety. Mathematical modeling can be used to make better use of more shallow data and to extract information from it with higher efficiency. Cultivars of chickpea, *Cicer arietanum*, are currently being improved by introgressing wild *C. reticulatum* biodiversity with very different flowering time requirements. More understanding is required for how flowering time will depend on environmental conditions in these cultivars developed by introgression of wild alleles.

**Results:**

We built a novel model for flowering time of wild chickpeas collected at 21 different sites in Turkey and grown in 4 distinct environmental conditions over several different years and seasons. We propose a general approach, in which the analytic forms of dependence of flowering time on climatic parameters, their regression coefficients, and a set of predictors are inferred automatically by stochastic minimization of the deviation of the model output from data. By using a combination of Grammatical Evolution and Differential Evolution Entirely Parallel method, we have identified a model that reflects the influence of effects of day length, temperature, humidity and precipitation and has a coefficient of determination of *R*^2^=0.97.

**Conclusions:**

We used our model to test two important hypotheses. We propose that chickpea phenology may be strongly predicted by accession geographic origin, as well as local environmental conditions at the site of growth. Indeed, the site of origin-by-growth environment interaction accounts for about 14.7% of variation in time period from sowing to flowering. Secondly, as the adaptation to specific environments is blueprinted in genomes, the effects of genes on flowering time may be conditioned on environmental factors. Genotype-by-environment interaction accounts for about 17.2% of overall variation in flowering time. We also identified several genomic markers associated with different reactions to climatic factor changes. Our methodology is general and can be further applied to extend existing crop models, especially when phenological information is limited.

**Electronic supplementary material:**

The online version of this article (10.1186/s12870-019-1685-2) contains supplementary material, which is available to authorized users.

## Background

Chickpea (*Cicer arietinum* L.), is the second most cultivated grain legume crop, grown in more than 50 countries of the world (ICARDA). Chickpea, which was originally domesticated in Southeastern Turkey, has been adapted to various environmental and climatic conditions across the globe from subtropical conditions in South Asia and East Africa to Northern regions of temperate North America. The time duration for chickpea to reach its reproductive phase is often limited by changing temperatures, rainfall pattern, daylength or competition for use of land by other crops in rotation [1, 2]. In Mediterranean and temperate regions, chickpeas are sown in spring where the day length and temperature increase towards the reproductive period, while in subtropical regions (Center and Southern India, Ethiopia, Queensland Australia) it is planted in the start of the dry season after the monsoonal rainy season when daylengths tend to be shorter and temperatures cooler. In the more temperate northern parts of India, the reproductive phase of spring-planted chickpea coincides with decreasing temperature and day length, whereas in the southern and the central parts of the country it falls within terminal drought (the end of the dry season) [3, 4]. Hence, chickpea breeding has focused on developing varieties differing in their growth duration to be able to adapt to different latitudes and sowing regimes [3, 5–7]. To achieve consistent yield, crop duration must closely match the available growing season [8]. Chickpea cultivars and landraces become increasingly temperature responsive as from the Mediterranean through northern, central and southern India, because these disparate origins have selected for contrasting phenological regulators [3]. This information is invaluable for modeling crop performance. For example, Vadez et al. (2012, 2013) [9–11] considered climatic factors like expected rainfall to predict performance of chickpeas in different geographical locations.

Several successful plant models like SSM [10, 12], DSSAT [13–17], APSIM [18] and others [19, 20] have been developed for legumes. These models use differential equations to describe biophysical and biochemical processes like photosynthesis, water uptake etc. and account for impact of genotype, soil, weather and economic factors. The influence of weather conditions is assessed using concepts like Heat Unit Index (HUI) [20], Crop Heat Units (CHI), Degree Days (DD), Biological Days (BD) [9] – all of them quantitatively characterizing the rate of progression to the next phenological phase on a daily basis. Both DD and BD could depend on temperature, water content and photoperiod. This formalism was applied to develop individual models for important crops. For example, DSSAT was used to simulate growth and yield in soybean [21] and chickpea [22] among several other crops [23–26]. For more than three decades these models have been applied in research projects of different countries. The SSM model was successfully tested using independent data from a wide range of growth [10, 12] and environmental conditions including Iran [27] and water deficit in India [11]. Considerable manipulations are required to adapt the DSSAT model to new environments and cultivars [28–32], limiting the utilization of these models. As varieties are constantly changing because of new releases that can cope with emerging pathogens and pests, as well as shifting consumer demands, the need for flexible models that can adjust to new varieties is high.

In an era of rapidly advancing genomic technologies and approaches, updated modeling approaches that can be tailored to genotype-specific effects are essential. Next generation sequencing and high throughput genotyping lead to identification of thousands of molecular markers (SSR, SNP, STMS, ESTs, CISP, DArT) [33] making it possible to construct chickpea genetic maps [34, 35] and ultimately to dissect the effect of different loci on key traits like flowering time. A combination of Sanger, 454/FLX and Illumina reads have been used to generate in transcriptome and genome assemblies for chickpea [34, 36–38].

Due to these advances in sequencing technologies and data acquisition, the genome-wide association study (GWAS) has become an important approach to understand the genetics of natural variation and traits of agricultural importance. Recent examples of GWAS in agriculturally important plants include identification of photoperiodic flowering time genes in sorghum [33], frost tolerance genes in barley (*Hordeum vulgare* L.) [34]; leaf architecture [35] and resistance to southern leaf blight genes in maize [36] as well as several agricultural traits in rice [37], to name a few. To extend GWAS to the analysis of genotype-by-environment (*G*×*E*) interactions bioclimatic variables can be used as a GWAS phenotype. Association between bioclimatic variables at a site of an accession’s origin and SNPs can indicate climatic adaptation [39]. While GWAS is a good method to identify genomic regions associated with important traits, typical GWAS designs require controlled planting of replicated accessions. This can quickly become logistically daunting and expensive across many sites.

Crop models may complement GWAS approaches by accounting for the influence of environmental factors [16]. However the models developed in the pre-genomic era considered genotype influence at best as a set of given “genetic coefficients” that do not correspond to actual genes [40]. Therefore these models were unable to simulate gene-by-environment interactions, thereby limiting their utility in predicting phenological characteristics of cultivars across different geographical locations and genotypes [9]. Mathematical models and tools that combine genetic and climate data to predict agronomic traits will greatly benefit breeders by simulating the performance of any given well-characterized genotype in any given well-characterized environment [41, 42].

While some of the current crop models consider the influence of local environmental conditions and others global climate changes for locally grown varieties, here, we built a new model using the flowering time of two species of wild chickpeas (*Cicer reticulatum* L. and *C. echinospermum*) collected at 21 different sites in Turkey and grown in 4 distinct environmental conditions. We further use our model to test two important hypotheses. Firstly, we propose that besides local environmental factors, chickpea phenology may be strongly predicted by accession geographic origin. Secondly, as the adaptation to specific environments is blueprinted in genomes, the effects of genes on flowering time may be conditioned on environmental factors. We check these hypotheses by statistical modeling of chickpea responses to climate change scenarios conditional on geographic site of origin and genotype.

## Materials and methods

### Dataset of wild chickpea accessions

**Fig. 1:**
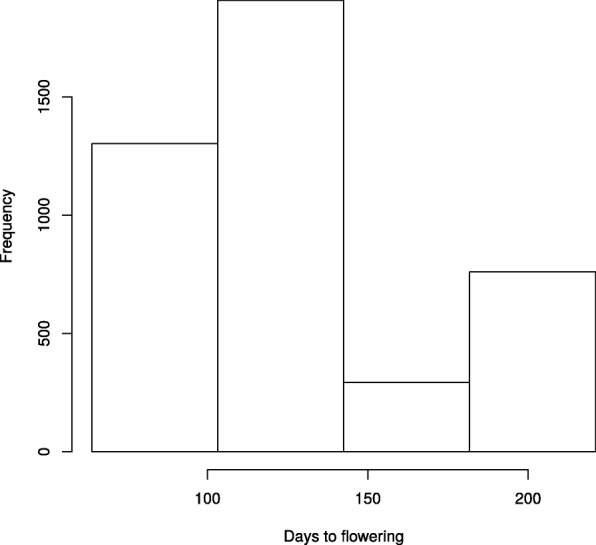
Distribution of time to flowering for the whole dataset. The range for time to flowering is from 64 to 221 day

The dataset consists of wild chickpea (*Cicer reticulatum* L. and *Cicer echinospermum*). Accessions were collected at 21 sites in five regions in Turkey (see Additional file 1: Table S1) by von Wettberg et al. [43]. These wild accessions were planted in climatically distinct sites in Turkey (Sanliurfa and Ankara, autumn and spring-sowing) and Australia (Floreat, near Perth, WA and Mt.Barker, WA). Being grown in contrasting environments the phenotype data on time to flowering is highly diverse. The distribution of time to flowering for the whole dataset is shown in Fig. 1. The time to flowering ranges from 64 to 221 day. Details on the phenotyping experiment and its subsequent analysis will be presented in the future manuscripts (Berger, J.: Analysis of phenotyping of wild chickpea in diverse environments, in preparation).

Climatic data was downloaded from NNDC Climate Data on-line [44]. The summary of agroclimatic factors as well as results of testing their correlation with flowering time are given in Additional file 1: Table S2 and S3, respectively.

A companion paper studying the genetic association of flowering time in one of the wild chickpeas (*Cicer reticulatum* L.) has identified six suggestive polymorphic sites associated with flowering time (Singh, A.: Genome-wide association studies in wild chickpea, in preparation). These SNPs were identified as the best SNPs after running a mixed linear model (MLM) in TASSEL, which associated flowering time (phenotype) with the genotypes using site/year/season as a factor to account for their effect on phenotype. Additional file 1: Table S6 presents number of times the reference allele for a SNP associated with flowering time is present in plant genotypes. To access genotype-environment interactions we group plants into 18 groups – one for each alternative (ALT) and reference (REF) allele combination (ALT/ALT, REF/ALT and REF/REF) – for each SNP and built a model (1) for each group.

### Regression model for time to flowering

We model a time period from sowing to flowering as a linear combination of *N* control functions *F*_*n*_, *n*=0,…,*N*−1 of agroclimatic factors. Thus, the model takes the form (1)

1 $$ y_{i}=\beta_{0}+\sum_{n=0}^{N-1}\beta_{n+1}\cdot F_{n}(\mathbf{X}_{i})+\varepsilon_{i} \qquad i=0,\dots,I-1   $$

where *y*_*i*_ is modeled phenotype (time from sowing to flowering) for each plant *i* from a group of the size *I*, *β*_*n*_ are coefficients, *n*=0,…,*N*, that are to be found to minimize the discrepancy between data and model, **X**_*i*_ is a vector of agroclimatic factors and *ε*_*i*_ is a standard error. The number of coefficients is *N*+1 because *β*_0_ is an intercept.

In comparison with previous models in our approach control functions *F*_*n*_ are automatically composed in analytic form from the expressions of climatic factors. Thus, a wider range of non-linear dependencies between the phenotype and factors is explored (see “Analytic form of control function” on page 17).

To study the adaptation to environment of origin we represent collection sites as *L*=21 binary variables, where *l*=1,…,*L* enumerates locations: Baristepe1, Baristepe2, Baristepe3, Beslever, Cermik, Cudi, Cudi2, Dereici, Destek, Egil, Gunasan, Kalkan, Karabahce, Kayatepe, Kesentas, Ortanca, Oyali, Sarikaya, Savur1, Sirnak1, Siv-Diyar (see column 1 in Additional file 1: Table S1). For each plant enumerated with *i*=0,…,*I*−1 one of the *L* variables $d_{i}^{l}$ takes the value ’1’ to indicate collection site and others are ’0’. The interaction between control function and location is modeled by an additional term in the regression function that has the form of a weighted sum of *N*·*L* pairwise products of control functions *F*_*n*_ and each binary site variable $d_{i}^{l}$.

Consequently, a model with information about a collection site takes the form (2).

2 $$ y_{i}=\beta_{0}+\sum_{n=0}^{N-1}\beta_{n+1}\cdot F_{n}(\mathbf{X}_{i})+\sum_{n=0}^{N-1}\sum_{l=1}^{L}\zeta_{l\cdot N+n}\cdot F_{n}(\mathbf{X}_{i})\cdot d^{l}_{i}+\varepsilon_{i}   $$

where in addition to notations used in (1) new regression coefficients *ζ*_*l*·*N*+*n*_ define the influence of function *F*_*n*_ of climatic factors on phenotype of plants collected at site *l* so that condition *ζ*_*l*·*N*+*n*_≠0 points on plant adaptation to the site. As a result, this model makes it possible to regress a range of climatic variables describing the phenotyping site (e.g. day length, temperature, precipitation etc.) independently for each of our 21 collection sites.

We denote *K* number of SNP and *J*=3 combinations of alternative (ALT) and reference (REF) alleles ALT/ALT, ALT/REF and REF/REF by 0, 1, and 2, respectively. Then to include GWAS results into the model we define *J*·*K* groups of plants so that members of the same group have the same combination of alleles in one of the SNP positions. Thus we define a matrix *D* with the number of rows equal to the number of plants *I* and *J*·*K* columns. Then, the elements of matrix *D* are defined by (3). Thus, the form of the regression function adapts to the allele combination of a plant by changing the weights of control functions.

3 $$ {\begin{aligned} d^{3k+j}_{i} = \left\{\begin{array}{ll} 1 & \ \ \text{if in plant} ~i \text{ the combination for SNP}~ k ~\text{is}~ j\\ 0 & \ \ \text{otherwise} \end{array}\right.  \end{aligned}}  $$

Consequently, a model with genetic information takes the form (4).

4 $$ {\begin{aligned} y_{i}=\beta_{0}+\sum_{n=0}^{N-1}\beta_{n+1}\cdot F_{n}(\mathbf{X}_{i})+\sum_{n=0}^{N-1}\sum_{k=0}^{K-1}\sum_{j=0}^{J-1}\rho_{(3k+j)N+n}\cdot F_{n}(\mathbf{X}_{i})\cdot d^{3k+j}_{i}+\varepsilon_{i}  \end{aligned}}  $$

where in addition to notations used in (1) new regression coefficients *ρ*_(3*k*+*j*)*N*+*n*_, define the effect of genotype-by-climatic factor interaction.

### Analytic form of control function

In previous studies different forms of dependencies between phenotype and climatic factors have been considered [45–50]. For example, “segmented”, “beta”, “quadratic” and “dent-like” functions were considered in [10]. A product of quadratic functions of day length and mean temperature was used in iterative regression analysis (IRA) [51] to characterize a developmental speed per day. An interphase speed was calculated as a product of the effects of day length, water deficit and temperature in [52].

We propose a more general approach, in which the analytic form of a control function together with regression coefficients and a set of predictors are inferred automatically by stochastic minimization of the deviation of the model output from data. We use a combination of Grammatical Evolution (GE) [53, 54], LASSO [55] and Differential Evolution Entirely Parallel (DEEP) [56, 57] method to recover analytic form of *F*_*n*_, find regression coefficients and determine the set of climatic factors, respectively [58]. Differential Evolution was proposed by Storn and Price in 1995 [59] as a heuristic stochastic optimization method. DEEP was developed by us for application in the field of bioinformatics [56]. It includes several recently proposed enhancements [57, 60]. More details can be found in Additional file 1: Section S5.

In GE, the analytic function form is built by decoding the sequence called “word” of *L* integers called codons. Decoding is performed according to simple rules of substitution that establish a correspondence between codons and either an elementary arithmetic operation: ‘+‘, ‘-‘, ‘*‘, ‘/‘, or expression: X, (X - Const), 1/(X - Const), where X is a name of a predictor and Const is some constant number (see Additional file 1: Section S2). To make estimation of regression coefficients with LASSO method reliable we performed 4-fold cross-validation at this stage so that the model was build using 75% of samples for training and the rest 25% was used for evaluation of the model.

### Statistical tests

We used standard statistical techniques for hypothesis testing implemented in R system for statistical computing [61]. We used multiple-way analysis of variance (MANOVA) with the Pillai test statistic [62] and ANOVA with the Fisher test statistic to check for significance in the difference of effects of climatic factors on phenotype between locations and genotypes. For pairwise comparison of the influence of climatic factors on phenotype between genotypes and locations we applied the Wilcoxon-Mann-Whitney test.

The Spearman’s rank correlation was used to estimate correlation between allele frequencies and climatic factors at primary collection sites (geographic sites of origin).

### Software tools

Although a few Grammatical Evolution (GE) implementations are freely available (see e.g. [54, 63]) they either lack a specific set of expressions or show low performance in experimental runs due to interpreted language (data not shown). Consequently a decision was made to implement GE in C++ using Armadillo [64], mlpack [65], HDF5 [66], HighFive [67] and Qt [68] as these packages provide efficient matrix operations, the LASSO method, data input-output and utility functions, respectively. The code is open-source on GitLab [69].

TASSEL (Trait Analysis by aSSociation, Evolution and Linkage) [70] was developed in Java, and is compatible with multiple operating systems (Windows, Linux and Mac OS). TASSEL can implement several different GWAS models like general linear model (GLM) and MLM using a GUI or command line version of the software.

## Results

We first performed ANOVA test to check for differences in mean time to flowering between accessions collected at different sites in Turkey to demonstrate that flowering time is an adaptive trait in chickpea. The difference in means was significant with criterion value *F*=2.003 and *p*=0.005<0.05.

### Model with interactions between climatic factors and locations

Next, to estimate the effect of interaction between climatic factors at phenotyping sites and sampling locations in Turkey on flowering time we built a model (2). A series of numerical experiments were performed, as several runs are needed to obtain a reliable solution with stochastic optimization. By several trial-and-error attempts (data not shown) it was established that the number of control functions *N*=12 and the length of the “word” *L*=5 were the best parameters for the model. The population size for DEEP was set to 500.

We obtained several solutions with coefficient of determination >0.85 and different analytic forms of the control functions (data not shown). We selected the model (5) as it reflects the influence of effects of day length, temperature, humidity and precipitation in the phenotyping environment and has coefficient of determination *R*^2^=0.97.

5 $$\begin{array}{*{20}l} {\mathtt{TTF}} =& 59.49 + 74.95 D^{min}_{x10} + 19.83/\left(T^{min}_{x5} - 0.03\right) - 1.98 P^{mean}_{x10} \\ & - 53.18 \left(D^{mean}_{x50} + 1/\left(U^{mean}_{x10-15} - 23.31\right)\right) - 13.04 D^{mean}_{x10-15} \\ &- (0.05 \cdot\mathtt{Baristepe1} + 0.12 \cdot\mathtt{Baristepe3} + 0.29 \cdot{\mathtt{Beslever}} \\ & + 0.31 \cdot{\mathtt{Dereici}} + 0.45 \cdot{\mathtt{Kayatepe}} + 0.03 \cdot{\mathtt{Kesentas}} \\ & + 0.74 \cdot\mathtt{Siv-Diyar} + 0.01 \cdot{\mathtt{Sarikaya}} + 0.10 \cdot\mathtt{Sirnak1} \\ & + 0.20 \cdot{\mathtt{Oyali}})\cdot \left(D^{mean}_{x50} + 1/\left(U^{mean}_{x10-15} - 23.31\right)\right) \\ & + (0.03 \cdot\mathtt{Cudi2} + 0.16 \cdot{\mathtt{Destek}} + 0.09 \cdot{\mathtt{Gunasan}}\\ & + 0.46 \cdot{\mathtt{Kesentas}} + 0.43 \cdot{\mathtt{Oyali}} \\ & + 0.28 \cdot\mathtt{Sirnak1})\cdot D^{min}_{x10} \\ & + (0.41 \cdot{\mathtt{Cudi}} + 0.05 \cdot{\mathtt{Karabahce}} - 0.003 \cdot{\mathtt{Kesentas}} \\ & + 0.02 \cdot{\mathtt{Oyali}} - 0.12 \cdot\mathtt{Sirnak1})\cdot D^{mean}_{x10-15},  \end{array} $$

where $D^{min}_{x10}$, $D^{mean}_{x50}$, $D^{mean}_{x10-15}$ denote minimum day length over 10 days after sowing, mean day length over a period of 50 days and from 10 to 15 days after sowing, respectively, $T^{min}_{x5}$ denotes minimum temperature over 5 days after sowing, $U^{mean}_{x10-15}$ denotes mean relative humidity over an interval from 10 to 15 days after sowing and $P^{mean}_{x10}$ denotes average precipitation over 10 days after sowing.

The analysis of relative difference in the sum of squares for a model with and without a term describing an interaction between climatic factor at the phenotyping site and the accession geographic site of origin allows us to conclude that sampling collection site-by-phenotyping environment interaction accounts for about 14.7% of variation in time period from sowing to flowering.

We found that day length-by-collection site interaction is important for locations Baristepe3, Cudi, Cudi2, Destek, Gunasan, Karabahce, and both day length and humidity-by-collection site interaction are important for Baristepe1, Beslever, Dereici, Kayatepe, Kesentas, Oyali, Siv-Diyar, Sarikaya, and Sirnak1 sampling sites. There were no interactions between climatic factors and collection sites in Baristepe2, Cermik, Egil, Kalkan, Ortanca and Savur1.

### Basic flowering time models for locations

To analyze how climatic factors at phenotyping sites affect flowering time of plants collected at different locations we built basic models (1) for groups of plants sampled at each location separately. We present selected models with the highest coefficients of determination (*R*^2^) between simulated and observed flowering time for each group in Additional file 1: Section S3. The distributions of time to flowering for these groups are presented in Additional file 1: Figure S2. Due to the stochastic nature of the procedure ten runs were performed with the same algorithmic parameters using different seeds for the random number generator to obtain an ensemble of models. Various factors and their combinations were selected as predictors by stochastic optimization.

Consequently, the effect of phenotyping environment day length, temperature, precipitation, humidity and their pairwise combinations on flowering time for plant groups was estimated with a coefficient of determination averaged over the ensemble of models, taking into account only terms dependent on the factor in question. The resulting coefficient values for each factor and factor combination are presented for all collection sites in Additional file 1: Figure S3–S7.

We compared the mean effect of climatic factors or factor combinations between accessions from different locations in Turkey with multiple-way ANOVA (MANOVA) using the values of coefficients of determination obtained in model runs as dependent variables and location as an independent variable. A Pillai statistics of 1.697 with *p*=0.037<0.05 confirmed the statistical significance of differences in mean influences of factors and their combinations on phenotype between locations.

Further, we applied one-way ANOVA to test the difference in effects on flowering time between accessions from different locations for each climatic factor or factor combination individually. Temperature, precipitation and their combination showed significant differences in the means of coefficient of determination values with *F*=3.617, *p*=1.806*e*−06 and *F*=2.233, *p*=0.003 and *F*=2.038, *p*=0.008 respectively.

### Analysis of the climatic factor effect on phenotype

**Fig. 2:**
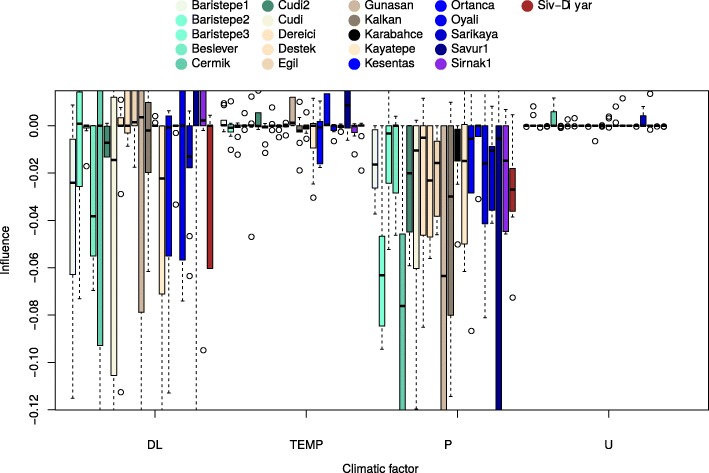
Analysis of climatic factor effects on phenotype. Box plots of climatic factor influence estimators calculated for model ensembles and for each location as a finite difference approximation of the partial derivative of a regression function (1) in respect to the factor. Each box covers two quantiles from 25 to 75% of influence’s variation with a horizontal line at median value of the estimated influence. Empty circles represent outliers. Boxes located higher than zero mark on vertical axis represent a positive influence of a factor on flowering time. In this case increasing the factor speeds up flowering. Other boxes represent an opposite case. “DL”, “TEMP”, “P” and “U” correspond to factors related to day length, temperature, precipitation and humidity, respectively

We continue our analysis of effects of climatic factors on flowering time for accessions from different locations with a pair-wise comparison method. Firstly, the direction and extent of each factor influence on phenotype was estimated as a finite difference approximation of the partial derivative of a regression function (1) in respect to the factor. Figure 2 presents the box plots of factor influence estimators calculated for model ensembles and for each location.

It is evident that both effects of day length and temperature on flowering time are location-dependent. For accessions collected at some locations increasing day length (e.g. Egil) or temperature (e.g. Ortanca) speeds up the rate of flowering, while at other locations the response to these factors is reversed (e.g. Kesentas). Surprisingly there was a consistent effect of precipitation across all accessions whereby higher precipitation reduced the time to flowering. This result is consistent with negative correlation between precipitation measures and time to flowering (see Additional file 1: Table S3). In comparison with precipitation the influence of humidity is opposite for most locations: the flowering time increases with rise of humidity. The influences of factor combinations are comparatively negligible.

**Fig. 3:**
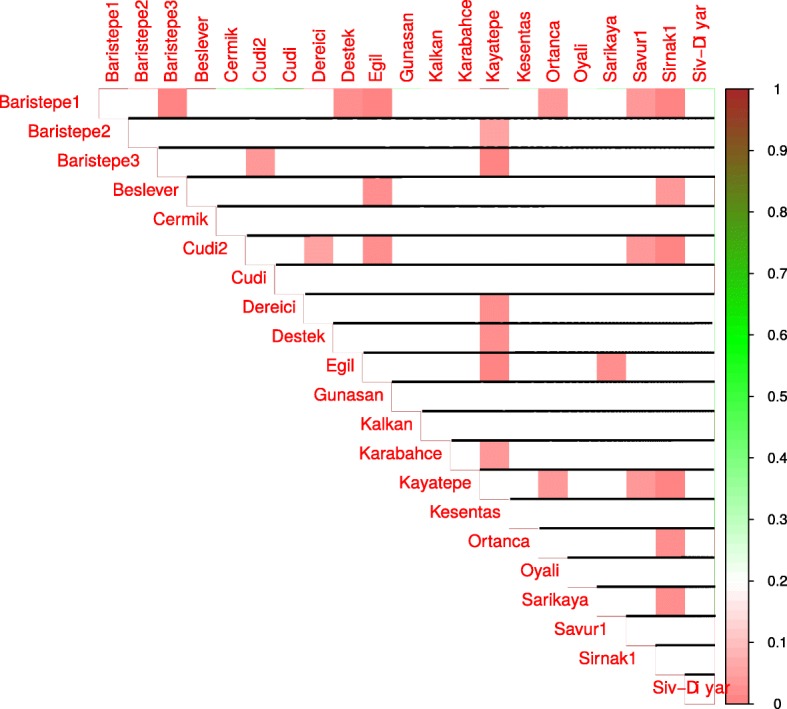
Results of pair-wise comparisons of day length influence on flowering time. Mann-Whitney-Wilcoxon test was applied to compare the means of day length influence estimators for locations. Statistically significant differences in means are shown as red color gradation, cells with statistically non-significant comparisons are left blank

**Fig. 4:**
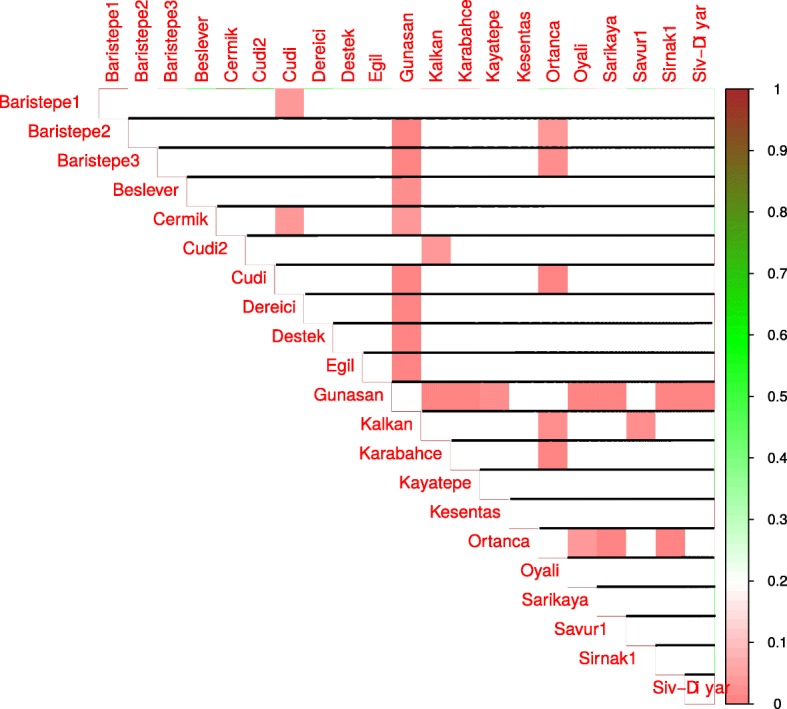
Results of pair-wise comparisons of temperature influence on flowering time. Mann-Whitney-Wilcoxon test was applied to compare the means of temperature influence estimators for locations. Statistically significant differences in means are shown as red color gradation, cells with statistically non-significant comparisons are left blank

**Fig. 5:**
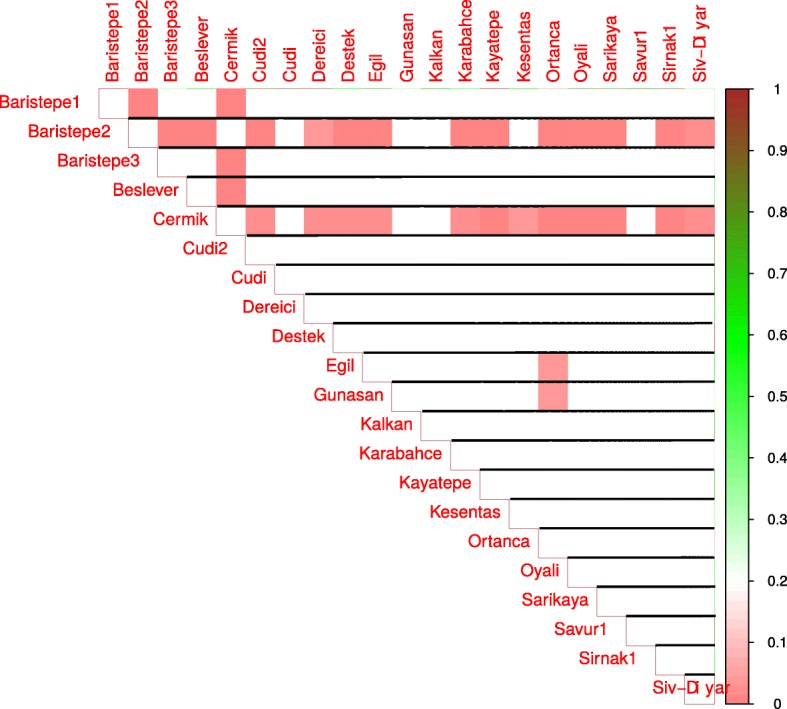
Results of pair-wise comparisons of precipitation influence on flowering time. Mann-Whitney-Wilcoxon test was applied to compare the means of precipitation influence estimators for locations. Statistically significant differences in means are shown as red color gradation, cells with statistically non-significant comparisons are left blank

**Fig. 6:**
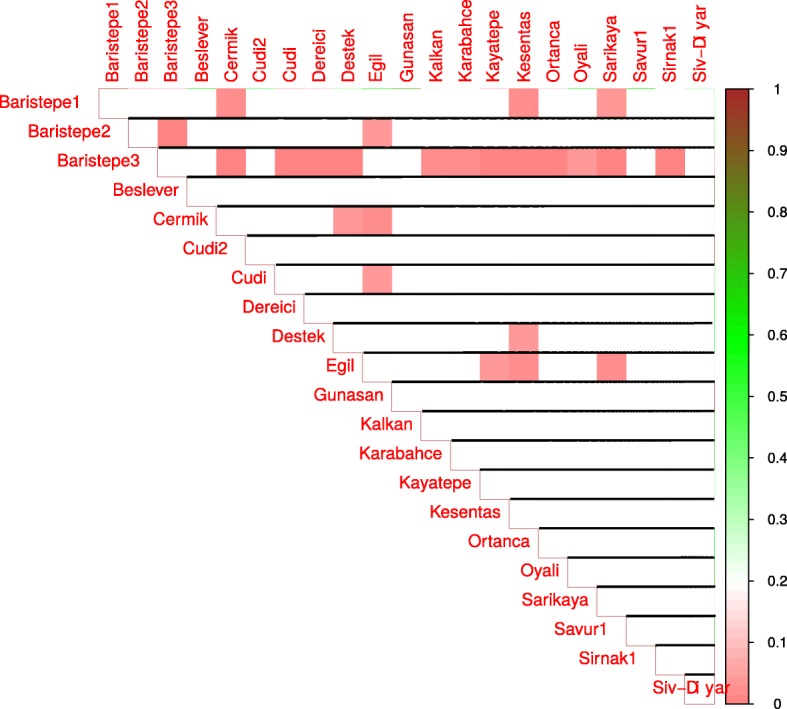
Results of pair-wise comparisons of humidity influence on flowering time. Mann-Whitney-Wilcoxon test was applied to compare the means of humidity influence estimators for locations. Statistically significant differences in means are shown as red color gradation, cells with statistically non-significant comparisons are left blank

Next, we compared the means of estimators of a factor’s influence on phenotype for location pairs with a Wilcoxon-Mann-Whitney test. Statistically significant differences in means between locations pairs are presented in Figs. 3, 4, 5, and 6 for day length, temperature, precipitation and humidity, respectively.

### Flowering time model with climatic factor-by-genotype interaction

Different genotypes may react differently to climatic factors. Here we check this hypothesis using the flowering time model (4) with the interaction term between climatic factors and genotype. We identified six SNPs associated with flowering time (see Additional file 1: Table S5). Here we subdivided all the plants into 18 groups, each containing similar allele combination at one of six polymorphic sites (see the “Regression model for time to flowering” section for more details). We further refer to these groups as SNP groups.

Ten runs were performed with the same algorithmic parameters but different seeds for random number generator. The model (6) with the best coefficient of determination *R*^2^=0.97 was selected for further analysis.

6 $$ {\begin{aligned} {\mathtt{TTF}} = & -5.71\cdot T^{max}_{x5-10} - 3.87\cdot T^{max}_{x15-20} - 0.39\cdot \left(1/\left(D^{min}_{x60} \,-\, 293.08\right) + T^{max}_{x10-15}\right) \\ + & 5.42\cdot D^{sum}_{x10-15}/\left(D^{min}_{x15-20} \cdot P^{mean}_{x50} + 1\right) \\ + & 20.08\cdot \left(T^{mean}_{x5-10} + \left(U^{mean}_{x10-15} - 0.004\right)/ \left(T^{min}_{x5-10} - 210.12\right)\right) \\ + & (0.06\cdot\mathtt{snp5AA} + 0.49\cdot\mathtt{snp3RR})\cdot T^{max}_{x5-10} \\ & + 0.05\cdot\mathtt{snp3AA}\cdot T^{max}_{x15-20}  \\ - & (0.02\cdot\mathtt{snp1RR} + 0.002\cdot\mathtt{snp2AA} + 0.16\cdot\mathtt{snp2RR} \\ & + 0.09\cdot\mathtt{snp3AA} + 0.50\cdot\mathtt{snp3RR} + 0.007\cdot\mathtt{snp4RR} \\ & + 0.14\cdot\mathtt{snp5RR} + 0.04\cdot\mathtt{snp6RR})\cdot \left(1/\left(D^{min}_{x60} - 293.08\right) + T^{max}_{x10-15}\right) \\ + & 0.11\cdot\mathtt{snp4RR}\cdot D^{sum}_{x10-15} /\left(D^{min}_{x15-20}\cdot P^{mean}_{x50} + 1\right) \\ + & 0.54\cdot\mathtt{snp3AA}\cdot T^{mean}_{x5-10}, \end{aligned}}  $$

where $D^{min}_{x60}$, $D^{min}_{x15-20}$ denote minimum day length over 60 days after sowing and over a period from 15 to 20 day after sowing, respectively; $D^{sum}_{x10-15}$ denotes sum of day lengths over a period from 10 to 15; $T^{max}_{x15-20}$, $T^{max}_{x10-15}$ and $T^{max}_{x5-10}$ denote maximum temperatures over a periods from 15 to 20, from 10 to 15 and from 5 to 10 days after sowing, respectively; $T^{mean}_{x5-10}$ and $T^{min}_{x5-10}$ denote mean and minimum temperatures over a period from 5 to 10 days after sowing, respectively; $U^{mean}_{x10-15}$ denotes mean humidity of an interval from 10 to 15 days after sowing and $P^{mean}_{x50}$ denotes mean precipitation of a period over 50 days after sowing.

While SNPs were identified only in *Cicer reticulanum* samples from 15 collection sites we are able to fit the model to the whole dataset giving appropriate values to the indicator variables – the elements of matrix *D* (see formulae 3 and 4).

The analysis of relative difference in the sum of squares for a model with and without the interaction terms between climatic factors and each SNP group allows us to conclude that genotype-by-environment interaction accounts for about 17.2% of variation in time period from sowing to flowering. All SNPs interact with temperature and day length. Additionally, SNP3 interacts with relative humidity and SNP4 interacts with precipitation.

To analyze the difference in response of SNP groups to climatic factors we built regression models (1) for each group separately. The distributions of time to flowering for these groups are presented in Additional file 1: Figure S8–S13. Selected models are presented in Additional file 1: Section S4.

Due to the stochastic nature of the procedure ten runs were performed with the same algorithmic parameters using different seeds for the random number generator to obtain an ensemble of models. Various agroclimatic factors and their combinations were selected as predictors by stochastic optimization.

We calculated the coefficients of determination for ensemble of models from which the terms that do not contain a predictor of a climatic factor or a combination of factors analyzed were excluded. The box plots of coefficient values for day length, temperature, precipitation, humidity and their combinations are presented in Additional file 1: Figure S14–S19 for all SNPs.

The multiple-way ANOVA (MANOVA) applied to the coefficient of determination as dependent variable and SNP group membership as an independent variable showed that the difference in mean effects of climatic factors on SNP groups is statistically significant (Pillai satistic value 1.287, *p*=1.333*e*−09<0.05).

Next we applied one-way ANOVA to test the influence of each climatic factor individually. The significant differences in the means of the coefficient determination values were observed for day length, humidity and the combination of precipitation and day length (*F*=2.102, *p*=0.009497 and *F*=6.642, *p*=5.159*e*−12 and *F*=1.904, *p*=0.0218 respectively).

The next step in our analysis was the pair-wise comparison of climatic factor influences on flowering time between SNP groups. The direction and extent of each factor influence on phenotype was estimated as a finite difference approximation of the partial derivative of a regression function in respect to the factor. Additional file 1: Figure S20 presents the box plots of factor influence estimators calculated for model ensembles and for each SNP group.

The means of estimators of a factor influence on phenotype averaged over SNP groups were compared with a Mann-Whitney-Wilcoxon test. As is evident from an analysis of Table 1, climatic factors had divergent effects on genotypes with different reference alleles at five out of six polymorphic position analyzed. As an example, for SNP1 (T →G) day length has different effects on plants with ALT/ALT and REF/ALT, as well as REF/REF and ALT/ALT allele combinations. Precipitation influences plants with ALT/ALT and REF/REF combinations differently. In case of SNP2 (A →G) we found clear differences between genotypes with ALT/ALT and REF/REF for combination of day length with either temperature or precipitation. For SNP3 (C →T) humidity affects genotypes with ALT/ALT and REF/REF differently, day length – temperature combination exerts different influence on ALT/ALT and ALT/REF genotypes, as well as ALT/REF and REF/REF genotypes, day length – precipitation combination shows different effects on ALT/ALT and REF/ALT genotypes. For SNP5 (C →A) there is difference in influence of day length on REF/REF and REF/ALT, as well as REF/ALT and ALT/ALT genotypes. In addition, precipitation also affects differently ALT/REF and REF/REF genotypes. Different effects of day length on ALT/ALT and REF/ALT genotypes is evident for SNP6 (A →G).

**Table 1:** Statistically significant differences in effects of climatic factors and their combinations on plant genotype

SNP	Factor	Genotype pairs	*P* value
1	DL	REF/ALT vs. REF/REF	0.035
1	P	ALT/ALT vs. REF/REF	0.032
1	DL	ALT/ALT vs. REF/REF	0.044
2	DL*TEMP	ALT/ALT vs. REF/REF	0.021
2	DL*P	ALT/ALT vs. REF/REF	0.040
3	DL*TEMP	ALT/ALT vs. REF/ALT	0.017
3	U	ALT/ALT vs. REF/REF	0.019
3	DL*TEMP	REF/ALT vs. REF/REF	0.045
3	DL*P	ALT/ALT vs. REF/ALT	0.049
5	DL	ALT/ALT vs. REF/ALT	0.042
5	DL	REF/ALT vs. REF/REF	0.043
5	P	REF/ALT vs. REF/REF	0.021
6	DL	ALT/ALT vs. REF/ALT	0.021

The pair-wise comparisons were performed with Mann-Whitney-Wilcoxon test. DL- day lengthh, TEMP – temperature, P – precipitation; REF – reference allele, ALT – alternative allele for polymorphic site

**Fig. 7:**
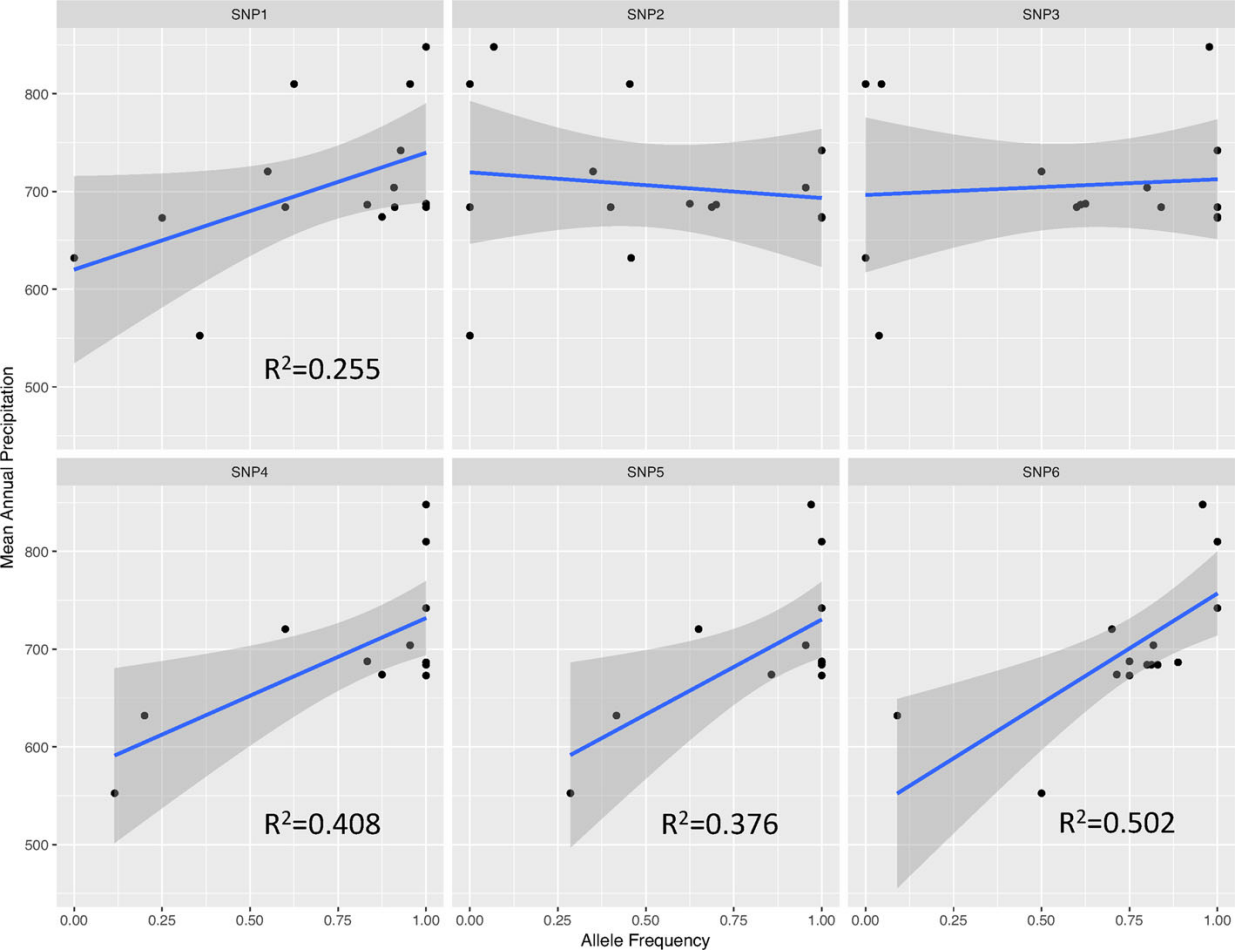
Correlations of mean annual precipitation (mean_annual_prec) with allele frequency of the 6 GWAS SNPs calculated for 15 populations of the wild chickpeas (shown for completeness). Allele frequency of SNPs 1, 4,5 and 6 are correlated with mean annual precipitation. The allele frequencies have a linear relationship at each of these significant SNPs, showing that these alleles are nearly fixed in the population in regions with high mean annual precipitation

To further understand the relationship between precipitation and the allele frequency of the SNPs, we correlated the allele frequency of 15 populations (see Additional file 1: Table S4) at the putative GWAS SNPs with the mean annual precipitation at the primary collection sites of the genotypes. Allele frequency of the SNPs 1, 4, 5 and 6 have a linear relationship and are correlated with mean annual precipitation. This is indicative of the alleles being fixed in the populations which are found in the areas with high mean precipitation (see Fig. 7). Spearman’s rank correlation between mean annual temperature and the allele frequency of the SNPs resulted in no significant relationship. This is indicative of 2 possible scenarios, a) the mean annual temperature value might not be indicative of critical time window affecting the time of flowering in the genotypes, b) the SNP alleles are not in genes involved in the pathways of temperature response (see Additional file 1: Figure S1).

## Discussion

The lifecycle of chickpea is strongly determined by environmental factors. Consequently, its phenology is likely strongly predicted by geographic origin and local phenotyping environment, as demonstrated in domestic chickpea cultivars and landraces originating from the Mediterranean to southern India [3]. Here we investigate this hypothesis in the wild progenitors of chickpea by statistical modeling of chickpea responses to environment conditional on geographic site of origin and genotype. Usually the extent of *G*×*E* interaction due to sampling site and environmental factors is modeled by state-of-the-art techniques such as AMMI and factorial regression or by using bioclimatic variables as a GWAS phenotype. Here we implemented a more general solution in which the analytic form of dependencies between predictors (climatic factors, collection sites and genotypes) and phenotype (flowering time) is automatically inferred by a stochastic optimization technique. Apart from automation the advantage of our approach resides in its ability to quickly examine different fits to the data and select the optimal one. We performed model parameterization on a wild chickpea dataset collected at 21 different locations in Turkey [43] grown in 4 different environments. GWAS analysis of the data identified six polymorphic sites responsible for flowering time variation independent of environmental conditions (Singh, A.: Genome-wide association studies in wild chickpea, in preparation).

We built two types of flowering time models – for the whole dataset and for groups of plants, that either originated from one sampling site or have similar allele combination at one of the 6 SNP positions.

Using the models for the whole dataset we found that 14.7% and 17.2% of variation in time to flowering is accounted for by interactions of climatic factors with geographic origin of the plant and its genotype, respectively. Contrary to previous approaches that measure the combined sensitivity of the phenotype to all environmental factors, our approach makes it possible to identify responses to specific environmental conditions and sampling locations in individual accessions, collection sites or SNP groups. In this case we have treated collection site as a model parameter which describes the composite influence of geography (latitude, altitude etc.) climate (day length, temperature) and biological interactions on phenotype. We found that in total 15 out of 21 sampling sites interact with different climatic factors at the phenotyping site, day length and humidity in particular. We also showed that all of six polymorphic sites identified in GWAS interact with temperature and day length, and that SNP3 and SNP4 additionally interact with relative humidity and precipitation respectively.

The influence of the geographic site of origin on plant phenology was further confirmed by applying a group-oriented approach. We found that wild chickpea accessions originating from different collection sites react differently to different environments. For example, plants collected at Baristepe1 react differently to day length change in comparison to plants from locations Baristepe3, Destek, Egil, Ortanca, Savur1 and Sirnak1 (see Fig. 3).

Observing the relation between climatic factors at the site of genotype collection, we hypothesized that there should be an association between the allele frequency of the GWAS SNPs and climatic factors at genotype collection site. This was confirmed by strong correlations of allele frequency with collection site mean annual precipitation in 4 of the 6 SNP groups (Fig. 7). Three of these four SNPs, have fixed alleles (allele frequency 1) within populations with highest mean precipitation. This makes sense, given strong selection for climate-appropriate flowering time in Mediterranean annuals, which typically flower early to avoid terminal drought in low rainfall regions, but flower later to maximize their reproductive potential in longer season, high rainfall environments [71]. In this context we were interested to discover that there was no correlation of SNP allele frequencies with collection site annual mean temperature. This may be explained by strong site and SNP interaction for phenotyping temperature whereby genotypes collected at different locations responded differently to temperature (and precipitation). Thus, increasing temperature led to earlier flowering in some locations (e.g. Ortanca, Savur) and later flowering in others (e.g. Kesentas) (see Fig. 2), that makes it impossible to reveal dependencies between SNP frequencies and temperature with standard correlation analysis.

We were also able to demonstrate that certain environmental variables differently affect flowering time of genotypes with different allele combinations at five out of six polymorphic position analyzed. For example, different allele combinations at SNP1 differently react on day length change (see Table 1). This is an important characteristic of the SNP that might be used in practice.

We believe that the models we have developed here can be plugged into existing process-based models, such as SSM, to build a new generation of crop models that predicts aspects of crop performance based on genetic, geographic, environmental and management data. In an era of growing genomic information, these new models are essential. Specific subroutines modeling selected biological processes could be modified to incorporate effects on these variables without altering other processes within the model. With Grammatical Evolution and DEEP this can be achieved in automatic way, easing the adaptation of crop models in breeding programs around the world.

## Conclusions

Analyzing patterns of adaptation is a key for defining strategies to cope with GxE interactions in breeding for either wide or specific adaptation. The phenology of adaptive traits, like flowering time, may be strongly predicted by plant geographic origin and local environmental factors. Here we tested this hypothesis by statistical modeling of wild chickpea flowering time responses to different environmental conditions. Our results showed that 1) geographic origin of a plant is indeed a good predictor of flowering time in chickpea and 2) allele combinations at GWAS hits associated with flowering time are “environmentally responsive”, i.e. react differently to changes in climatic factors.

Our methodology is generic and can be further applied and extended to existing crop models.

## Additional file

Additional file 1 Additional file 1 contains information on SNP based groups, climatic data for these groups, details on Grammatical evolution method. (PDF 634 kb)

## Abbreviations

AMMI: Additive main effect and multiplicative interaction
APSIM: Agricultural Production Systems sIMulator
BD: Biological Days
CHI: Crop Heat Units
CISP: Conserved-intron scanning primers
DArT: Diversity Arrays Technology
DD: Degree Days
DEEP: Differential Evolution Entirely Parallel
DSSAT: Decision Support System for Agrotechnology Transfer
EST: Expressed Sequence Tags
GE: Grammatical Evolution
GLM: General linear model
GUI: Graphical user interface
GWAS: Genome-wide association studies
HUI: Heat Unit Index
ICARDA: International Center for Agricultural Research in the Dry Areas
IRA: Iterative regression analysis
LASSO: Least absolute shrinkage and selection operator
MANOVA: Multiple-way analysis of variance
MLM: Mixed linear model
SNP: Single nucleotide polymorphism
SSM: Simple Simulation Modeling
SSR: Single sequence repeat
STMS: Sequenced tagged microsatellite site
TASSEL: Trait Analysis by aSSociation, Evolution and Linkage
TTF: Time to flowering
