# Supplementary material for: Non-linear regression models for time to flowering in wild chickpea combine genetic and climatic factors
Source: BMC Plant Biol. 2019 Mar 19;19(Suppl 2):94. doi: 10.1186/s12870-019-1685-2 (PMC6423741; doi:10.1186/s12870-019-1685-2)
Supplement: Supplementary file 1 — Additional file 1 contains information on SNP based groups, climatic data for these groups, details on Grammatical evolution method. (PDF 634 kb) [file 12870_2019_1685_MOESM1_ESM.pdf]

Non-linear regression models for time to flowering in wild chickpea combine genetic and climatic factors. Supporting Information

Konstantin Kozlov, Anupam Singh,
Jens Berger, Eric Bishop-von Wettberg, Abdullah Kahraman, Abdulkadir Aydogan,
Douglas Cook, Sergey Nuzhdin, and Maria Samsonova

November 6, 2018

S1 Dataset and data analysis

Table S1: Number of genotypes collected at each geographic location (site) within each region. Rows are site names, columns – region names.

L	Name	Adiyaman	Diyarbakir	Mardin	Sirnak	Urfa
1	Baristepe1	0	0	279	0	0
2	Baristepe2	0	0	162	0	0
3	Baristepe3	0	0	341	0	0
4	Beslever	0	0	225	0	0
5	Cermik	0	117	0	0	0
6	Cudi	0	0	0	256	0
7	Cudi2	0	0	0	270	0
8	Dereici	0	0	250	0	0
9	Destek	0	0	0	0	229
10	Egil	0	241	0	0	0
11	Gunasan	0	90	0	0	0
12	Kalkan	0	171	0	0	0
13	Karabahce	0	0	0	0	363
14	Kayatepe	0	0	172	0	0
15	Kesentas	0	134	0	0	0
16	Ortanca	0	0	0	0	54
17	Oyali	165	0	0	0	0
18	Sarikaya	0	0	242	0	0
19	Savur 1	0	0	35	0	0
20	Sirnak 1	0	0	0	307	0
21	Siv-Diyar	0	0	0	0	159

Table S2: The summary of agroclimatic factors. D, T, U and P denote day length, temperature, relative humidity and precipitation, respectively. Superscripts `min`, `max` and `mean` denote minimum, maximum and mean values of a factor over a period of time defined in subscript where a number preceeding or succeeding `x` defines a day before or after sowing, respectively. Superscript `flowering10` denotes a factor value at a day when 10% of plants flower.

Symbol	Adiyaman	Diyarbakir	Mardin	Sirnak	Urfa
$T^{mean}_{sowing-flowering}$	13.8+-1.09	13.81+-1	13.42+-0.88	13.78+-0.89	13.71+-1.06
$T^{sum10}_{sowing-flowering}$	22324.39+-4299.09	22009.61+-3951.83	19678.2+-4228.83	20880.42+-4626.72	20979.63+-5182.72
$T^{sum15}_{sowing-flowering}$	13095.12+-4536.61	13020.19+-4206.99	11266.9+-4043.06	12658.34+-4226.95	12565.54+-5285.44
$T^{sum}_{sowing-flowering}$	25588.91+-4167.94	25195.35+-3810.02	22803.86+-4126.22	23992+-4559.97	24077.34+-4956.23
$T^{max}_{sowing-flowering}$	29.68+-3.5	29.13+-3.53	27.27+-3.49	29.92+-2.54	27.96+-3.8
$T^{min}_{sowing-flowering}$	0.8+-1.21	0.77+-1.35	0.39+-1.32	0.31+-1.27	0.38+-1.33
$U^{mean}_{sowing-flowering}$	63.19+-10.49	62.36+-10.16	63.19+-9.1	61.2+-9.01	62.69+-9.54
$P^{mean}_{sowing-flowering}$	8.06+-1.06	7.86+-1.18	7.82+-1.23	7.88+-1.16	7.86+-1.24

$P^{sum}_{sowing-flowering}$	14940.97+-2953.69	14348.83+-3015.66	13256.59+-2992.68	13669.27+-3073.65	13644.53+-2650.27
$D^{sum}_{sowing-flowering}$	21704.27+-3496.53	21529.24+-3423.05	20118.88+-3634.03	20848.89+-3914.34	20911.95+-4438.98
$D^{max}_{sowing-flowering}$	13.37+-0.71	13.4+-0.76	13.36+-0.84	13.59+-0.71	13.41+-0.9
$D^{min}_{sowing-flowering}$	10.42+-0.71	10.52+-0.82	10.62+-0.91	10.62+-0.86	10.63+-0.92
$D^{mean}_{sowing-flowering}$	11.72+-0.89	11.81+-0.98	11.87+-1.07	12+-0.98	11.92+-1.11
T^{sowing}	10.95+-3.85	11.07+-3.95	10.61+-3.6	10.35+-3.51	10.61+-3.6
U^{sowing}	57.41+-12.49	57.04+-12.65	57.92+-11.85	57.58+-11.35	57.93+-11.86
P^{sowing}	8.39+-2.03	8.35+-2.01	8.39+-2.02	8.27+-1.97	8.4+-2.02
D^{sowing}	10.77+-0.89	10.77+-0.87	10.8+-0.95	10.84+-0.99	10.8+-0.95
T^{mean}_{5x5}	11.54+-3.69	11.63+-3.81	11.23+-3.38	10.98+-3.23	11.24+-3.38
T^{max}_{5x5}	19.95+-3.06	20.12+-3.13	19.5+-2.85	19.21+-2.75	19.5+-2.85
T^{min}_{5x5}	3.33+-4.59	3.43+-4.7	2.99+-4.3	2.68+-4.18	2.99+-4.3
U^{mean}_{5x5}	64.2+-11.23	63.32+-11.38	66.12+-10.39	66.7+-9.72	66.12+-10.4
P^{mean}_{5x5}	7.24+-1.76	7.27+-1.76	7.11+-1.74	6.96+-1.73	7.11+-1.75
D^{sum}_{5x5}	2329.78+-605.95	2334.14+-597.86	2335.83+-624.76	2363.19+-638.04	2335.16+-624.71
D^{max}_{5x5}	10.93+-0.94	10.93+-0.91	10.95+-1	11+-1.03	10.95+-0.99
D^{min}_{5x5}	10.62+-0.84	10.62+-0.82	10.65+-0.9	10.69+-0.94	10.65+-0.9
D^{mean}_{5x5}	10.77+-0.89	10.77+-0.87	10.8+-0.95	10.84+-0.99	10.8+-0.95
T^{mean}_{x10}	11.58+-3.16	11.67+-3.21	11.28+-2.99	11.01+-2.9	11.28+-2.99
T^{max}_{x10}	19.86+-3.15	20.04+-3.21	19.38+-2.96	19.07+-2.87	19.38+-2.96
T^{min}_{x10}	3.74+-3.63	3.78+-3.61	3.56+-3.67	3.33+-3.72	3.56+-3.67
U^{mean}_{x10}	66.5+-11.82	65.66+-11.98	68.31+-11.02	68.83+-10.42	68.31+-11.03
P^{mean}_{x10}	7.28+-1.99	7.31+-1.98	7.11+-1.97	6.89+-1.89	7.12+-1.97
D^{sum}_{x10}	2326.88+-625.96	2329.83+-618.27	2336.81+-644.1	2366.64+-657.41	2336.1+-644.05
D^{max}_{x10}	10.91+-1.02	10.91+-1	10.95+-1.08	11+-1.13	10.95+-1.08
D^{min}_{x10}	10.62+-0.92	10.62+-0.9	10.66+-0.98	10.7+-1.03	10.66+-0.98
D^{mean}_{x10}	10.77+-0.97	10.76+-0.95	10.8+-1.03	10.85+-1.08	10.8+-1.03
T^{mean}_{10x}	11.48+-3.98	11.58+-4.11	11.15+-3.63	10.88+-3.46	11.15+-3.63
T^{max}_{10x}	20.19+-4.33	20.32+-4.38	19.77+-4.18	19.4+-4.11	19.77+-4.18
T^{min}_{10x}	3.33+-4.96	3.44+-5.11	2.96+-4.58	2.65+-4.41	2.96+-4.58
U^{mean}_{10x}	63.93+-9.09	63.32+-9.24	65.33+-8.46	65.84+-8.1	65.33+-8.47
P^{mean}_{10x}	7.43+-1.78	7.41+-1.78	7.41+-1.74	7.29+-1.69	7.41+-1.75
D^{sum}_{10x}	2218.76+-551.95	2243.08+-555.25	2167.29+-538.79	2154.64+-536.37	2167.24+-538.89
D^{max}_{10x}	10.95+-0.87	10.95+-0.85	10.97+-0.92	11.01+-0.96	10.97+-0.92
D^{min}_{10x}	10.65+-0.82	10.65+-0.8	10.68+-0.89	10.72+-0.93	10.68+-0.89
D^{mean}_{10x}	10.8+-0.85	10.8+-0.82	10.82+-0.91	10.86+-0.95	10.82+-0.91
T^{mean}_{x5}	12.09+-3.46	12.2+-3.52	11.76+-3.29	11.48+-3.21	11.76+-3.29
T^{max}_{x5}	19.86+-3.15	20.04+-3.21	19.38+-2.96	19.07+-2.87	19.38+-2.96
T^{min}_{x5}	4.92+-4.3	4.99+-4.31	4.6+-4.24	4.24+-4.2	4.61+-4.24
U^{mean}_{x5}	62.92+-12.65	61.99+-12.85	64.91+-11.6	65.42+-10.74	64.91+-11.62
P^{mean}_{x5}	7.37+-2.01	7.42+-2	7.16+-2.02	6.94+-2	7.16+-2.02
D^{sum}_{x5}	1266.34+-335.64	1267.71+-330.95	1272.28+-346.79	1288.7+-354.65	1271.9+-346.75
D^{max}_{x5}	10.84+-0.95	10.84+-0.93	10.87+-1.02	10.92+-1.06	10.87+-1.01
D^{min}_{x5}	10.69+-0.91	10.69+-0.88	10.72+-0.97	10.77+-1.01	10.72+-0.97
D^{mean}_{x5}	10.77+-0.93	10.76+-0.91	10.8+-0.99	10.84+-1.03	10.8+-0.99
T^{mean}_{x5-15}	10.8+-2.29	10.89+-2.28	10.47+-2.26	10.17+-2.18	10.48+-2.26
T^{max}_{x5-15}	19.4+-3.04	19.61+-2.96	18.84+-3.21	18.46+-3.32	18.84+-3.21
T^{min}_{x5-15}	2.55+-3.5	2.61+-3.44	2.26+-3.62	1.91+-3.64	2.26+-3.62
U^{mean}_{x5-15}	67.84+-12.92	66.98+-12.92	69.72+-12.63	70.33+-12.47	69.72+-12.64
P^{mean}_{x5-15}	7.2+-1.75	7.22+-1.75	7.06+-1.73	6.87+-1.65	7.07+-1.73
D^{sum}_{x5-15}	2338.02+-642.29	2341.75+-635.53	2346.34+-658.02	2375.79+-670.16	2345.63+-657.98
D^{max}_{x5-15}	10.91+-1.12	10.91+-1.1	10.95+-1.18	11.01+-1.23	10.95+-1.18
D^{min}_{x5-15}	10.63+-1.02	10.62+-0.99	10.68+-1.08	10.73+-1.12	10.67+-1.08
D^{mean}_{x5-15}	10.77+-1.07	10.76+-1.05	10.81+-1.13	10.87+-1.18	10.81+-1.13
T^{mean}_{x5-10}	11.18+-2.99	11.28+-3.02	10.85+-2.91	10.55+-2.85	10.85+-2.91
T^{max}_{x5-10}	17.96+-3.21	18.09+-3.23	17.55+-3.13	17.2+-3.08	17.55+-3.14
T^{min}_{x5-10}	4.89+-3.08	4.91+-3	4.79+-3.3	4.66+-3.47	4.79+-3.3
U^{mean}_{x5-10}	70.87+-12.88	70.09+-12.97	72.66+-12.46	73.31+-12.3	72.66+-12.46
P^{mean}_{x5-10}	7.04+-2.27	7.07+-2.24	6.84+-2.24	6.55+-2.09	6.84+-2.24
D^{sum}_{x5-10}	1262.94+-341.43	1263.71+-336.91	1270.46+-352.24	1287.78+-360.06	1270.06+-352.2
D^{max}_{x5-10}	10.84+-1.05	10.83+-1.02	10.87+-1.11	10.93+-1.15	10.87+-1.11
D^{min}_{x5-10}	10.69+-1	10.69+-0.97	10.73+-1.06	10.78+-1.1	10.73+-1.06
D^{mean}_{x5-10}	10.77+-1.02	10.76+-1	10.8+-1.08	10.86+-1.13	10.8+-1.08
T^{mean}_{x10-15}	10.38+-2.8	10.44+-2.76	10.13+-2.87	9.84+-2.87	10.13+-2.87
T^{max}_{x10-15}	17.91+-3.64	18.07+-3.6	17.43+-3.72	17.04+-3.77	17.43+-3.72
T^{min}_{x10-15}	3.61+-3.91	3.59+-3.86	3.48+-3.92	3.14+-3.81	3.48+-3.93
U^{mean}_{x10-15}	65.06+-14.14	64.15+-14.1	67+-13.89	67.52+-13.71	67+-13.9
P^{mean}_{x10-15}	7.37+-1.51	7.38+-1.5	7.29+-1.52	7.18+-1.52	7.29+-1.52
D^{sum}_{x10-15}	1290.32+-365.19	1293.87+-362.81	1291.28+-370.24	1305.83+-375.05	1290.91+-370.25
D^{max}_{x10-15}	10.84+-1.15	10.83+-1.12	10.89+-1.21	10.95+-1.26	10.88+-1.21
D^{min}_{x10-15}	10.71+-1.09	10.7+-1.07	10.75+-1.15	10.81+-1.2	10.75+-1.15
D^{mean}_{x10-15}	10.77+-1.12	10.76+-1.1	10.82+-1.18	10.88+-1.23	10.82+-1.18
T^{mean}_{x15-20}	10.73+-3.03	10.98+-3.01	10+-2.99	9.5+-2.91	10.01+-2.99
T^{max}_{x15-20}	19.54+-3.75	19.88+-3.72	18.67+-3.7	18.2+-3.68	18.68+-3.7
T^{min}_{x15-20}	2.76+-3.12	2.97+-3.15	2.15+-3	1.75+-2.92	2.16+-3
U^{mean}_{x15-20}	55.84+-14.66	54.61+-14.65	58.46+-13.93	59.14+-13.12	58.47+-13.95
P^{mean}_{x15-20}	7.65+-1.55	7.68+-1.56	7.51+-1.49	7.35+-1.44	7.51+-1.5
D^{sum}_{x15-20}	1291.95+-371.28	1295.24+-369.08	1293.62+-375.98	1308.64+-380.75	1293.24+-375.99
D^{max}_{x15-20}	10.86+-1.25	10.85+-1.23	10.91+-1.31	10.97+-1.36	10.91+-1.31
D^{min}_{x15-20}	10.73+-1.2	10.71+-1.18	10.78+-1.26	10.84+-1.31	10.78+-1.26
D^{mean}_{x15-20}	10.79+-1.22	10.78+-1.2	10.84+-1.28	10.9+-1.33	10.84+-1.28
T^{mean}_{x20-25}	11.88+-2.25	11.93+-2.28	11.74+-2.19	11.66+-2.2	11.74+-2.19
T^{max}_{x20-25}	19.92+-3.21	20.07+-3.17	19.52+-3.31	19.28+-3.42	19.52+-3.31
T^{min}_{x20-25}	4.5+-2.04	4.63+-2.08	4.18+-1.9	4.02+-1.83	4.18+-1.9
U^{mean}_{x20-25}	61.85+-12.71	61.38+-12.69	62.66+-12.59	62.54+-12.5	62.67+-12.6
P^{mean}_{x20-25}	7.54+-1.94	7.52+-1.96	7.5+-1.84	7.37+-1.74	7.51+-1.85
D^{sum}_{x20-25}	1293.18+-375.08	1296.57+-373.43	1294.53+-378.44	1309.29+-382.65	1294.16+-378.45
D^{max}_{x20-25}	10.89+-1.35	10.87+-1.33	10.94+-1.41	11.01+-1.46	10.94+-1.41
D^{min}_{x20-25}	10.76+-1.3	10.74+-1.28	10.81+-1.36	10.88+-1.41	10.81+-1.36
D^{mean}_{x20-25}	10.82+-1.33	10.81+-1.31	10.88+-1.39	10.94+-1.44	10.88+-1.39
T^{mean}_{x25-30}	11.8+-1.91	11.82+-1.96	11.76+-1.8	11.73+-1.77	11.76+-1.8
T^{max}_{x25-30}	18.64+-3.35	18.75+-3.38	18.42+-3.27	18.35+-3.29	18.42+-3.27

T^{min}_{x25-30}	4.89+-1.67	4.9+-1.75	4.89+-1.46	4.89+-1.36	4.89+-1.46
U^{mean}_{x25-30}	65.38+-9.68	65.12+-9.4	65.81+-10.33	65.66+-10.75	65.81+-10.33
P^{mean}_{x25-30}	7.31+-1.49	7.34+-1.5	7.23+-1.46	7.15+-1.47	7.23+-1.46
D^{sum}_{x25-30}	1238.89+-467.59	1248.99+-456.71	1220.32+-496.86	1220.48+-520.77	1220.17+-496.57
D^{max}_{x25-30}	10.92+-1.46	10.9+-1.44	10.98+-1.51	11.05+-1.57	10.98+-1.51
D^{min}_{x25-30}	10.8+-1.41	10.78+-1.39	10.86+-1.46	10.93+-1.52	10.86+-1.46
D^{mean}_{x25-30}	10.86+-1.43	10.84+-1.42	10.92+-1.49	10.99+-1.54	10.92+-1.49
T^{mean}_{x30-35}	11.3+-3.49	11.24+-3.41	11.5+-3.73	11.68+-3.94	11.5+-3.72
T^{max}_{x30-35}	20.24+-4.11	20.24+-4.04	20.36+-4.32	20.58+-4.49	20.35+-4.31
T^{min}_{x30-35}	2.53+-2.53	2.44+-2.52	2.85+-2.56	3.13+-2.58	2.84+-2.56
U^{mean}_{x30-35}	55.51+-15.67	54.82+-15.51	56.68+-15.66	56.35+-15.38	56.69+-15.67
P^{mean}_{x30-35}	7.44+-1.63	7.46+-1.62	7.33+-1.66	7.21+-1.7	7.34+-1.66
D^{sum}_{x30-35}	1335.75+-392.56	1332.57+-390.47	1357.93+-396	1390.44+-397.83	1357.28+-396.07
D^{max}_{x30-35}	11.09+-1.58	11.06+-1.56	11.23+-1.63	11.36+-1.68	11.23+-1.63
D^{min}_{x30-35}	10.96+-1.53	10.93+-1.51	11.09+-1.59	11.22+-1.65	11.09+-1.59
D^{mean}_{x30-35}	11.03+-1.56	10.99+-1.54	11.16+-1.61	11.29+-1.67	11.16+-1.61
T^{mean}_{x35-40}	11.71+-3.14	11.7+-3.03	11.62+-3.43	11.43+-3.63	11.62+-3.43
T^{max}_{x35-40}	19.28+-4.45	19.38+-4.23	18.89+-5.03	18.54+-5.42	18.9+-5.02
T^{min}_{x35-40}	3.36+-2.65	3.27+-2.62	3.6+-2.73	3.73+-2.84	3.6+-2.73
U^{mean}_{x35-40}	58.09+-9.71	57.58+-9.43	59.12+-10.28	59.24+-10.6	59.13+-10.28
P^{mean}_{x35-40}	7.53+-1.57	7.55+-1.57	7.41+-1.51	7.23+-1.43	7.42+-1.51
D^{sum}_{x35-40}	1352.18+-407.15	1350.19+-406.68	1371.34+-406.3	1402.35+-406.03	1370.7+-406.4
D^{max}_{x35-40}	11.15+-1.68	11.11+-1.66	11.29+-1.73	11.42+-1.79	11.29+-1.73
D^{min}_{x35-40}	11.02+-1.64	10.99+-1.62	11.16+-1.69	11.28+-1.75	11.15+-1.69
D^{mean}_{x35-40}	11.09+-1.66	11.05+-1.64	11.22+-1.71	11.35+-1.77	11.22+-1.71
T^{mean}_{x40-45}	11.03+-4.65	10.86+-4.56	11.51+-4.92	11.83+-5.17	11.51+-4.91
T^{max}_{x40-45}	18.21+-6.62	18.06+-6.58	18.63+-6.81	18.9+-7.09	18.62+-6.81
T^{min}_{x40-45}	3.27+-2.22	3.19+-2.19	3.54+-2.29	3.8+-2.33	3.54+-2.29
U^{mean}_{x40-45}	66.15+-14.65	65.96+-14.24	66.34+-15.81	66+-16.75	66.35+-15.8
P^{mean}_{x40-45}	6.5+-1.73	6.46+-1.75	6.52+-1.65	6.42+-1.57	6.52+-1.65
D^{sum}_{x40-45}	1278+-545.84	1267.78+-555.6	1317.36+-518.75	1357.39+-508.42	1316.65+-518.91
D^{max}_{x40-45}	11.21+-1.78	11.17+-1.76	11.35+-1.83	11.49+-1.89	11.35+-1.83
D^{min}_{x40-45}	11.09+-1.73	11.05+-1.71	11.23+-1.79	11.36+-1.85	11.23+-1.79
D^{mean}_{x40-45}	11.15+-1.75	11.11+-1.74	11.29+-1.81	11.42+-1.87	11.29+-1.81
T^{mean}_{x45-50}	11.29+-4.81	11.05+-4.79	11.92+-4.83	12.26+-4.95	11.91+-4.83
T^{max}_{x45-50}	19.3+-6.76	19+-6.74	20.01+-6.79	20.32+-6.96	20.01+-6.79
T^{min}_{x45-50}	3.55+-4.5	3.38+-4.54	4.1+-4.37	4.54+-4.36	4.09+-4.38
U^{mean}_{x45-50}	63.27+-14.46	62.87+-14.36	63.81+-14.62	63.41+-14.8	63.83+-14.63
P^{mean}_{x45-50}	7.14+-2.39	7.15+-2.37	6.98+-2.33	6.67+-2.16	6.98+-2.34
D^{sum}_{x45-50}	1418.92+-418.45	1422.19+-418.25	1426.65+-415.52	1454.04+-413.22	1426.01+-415.68
D^{max}_{x45-50}	11.47+-1.88	11.44+-1.87	11.58+-1.92	11.7+-1.98	11.58+-1.92
D^{min}_{x45-50}	11.34+-1.84	11.32+-1.83	11.45+-1.89	11.57+-1.94	11.45+-1.89
D^{mean}_{x45-50}	11.41+-1.86	11.38+-1.85	11.51+-1.91	11.63+-1.96	11.51+-1.91
T^{mean}_{x50-55}	11.78+-4.18	11.68+-4.25	12.02+-4.04	12.11+-4.06	12.02+-4.04
T^{max}_{x50-55}	20.23+-4.53	20.16+-4.51	20.36+-4.61	20.35+-4.77	20.36+-4.6
T^{min}_{x50-55}	4.23+-4.71	4.06+-4.77	4.77+-4.52	5.23+-4.44	4.76+-4.525
U^{mean}_{x50-55}	63.11+-12.77	62.22+-12.87	65.37+-12.31	66.55+-12.21	65.35+-12.31
P^{mean}_{x50-55}	7.18+-1.96	7.21+-1.92	6.98+-1.99	6.73+-1.95	6.99+-1.99
D^{sum}_{x50-55}	1420.57+-418.89	1423.34+-419.16	1429.29+-415.01	1456.82+-412.55	1428.65+-415.17
D^{max}_{x50-55}	11.55+-1.96	11.52+-1.95	11.66+-2	11.78+-2.06	11.66+-2
D^{min}_{x50-55}	11.43+-1.92	11.41+-1.91	11.54+-1.96	11.66+-2.02	11.54+-1.96
D^{mean}_{x50-55}	11.49+-1.94	11.46+-1.93	11.6+-1.98	11.72+-2.04	11.6+-1.98
T^{mean}_{x55-60}	11.23+-5.67	11.13+-5.72	11.44+-5.56	11.48+-5.64	11.44+-5.55
T^{max}_{x55-60}	18.48+-6.65	18.49+-6.56	18.48+-6.94	18.52+-7.29	18.48+-6.93
T^{min}_{x55-60}	4.55+-4.33	4.38+-4.39	4.98+-4.19	5.2+-4.2	4.98+-4.19
U^{mean}_{x55-60}	66.44+-13.57	65.88+-13.71	67.59+-13.13	67.82+-13.09	67.59+-13.13
P^{mean}_{x55-60}	7.38+-1.39	7.33+-1.37	7.45+-1.45	7.43+-1.5	7.45+-1.45
D^{sum}_{x55-60}	1294.66+-557.43	1304.73+-552.03	1278.02+-575.16	1282.04+-598.22	1277.79+-574.89
D^{max}_{x55-60}	11.64+-2.03	11.61+-2.02	11.75+-2.07	11.87+-2.13	11.75+-2.06
D^{min}_{x55-60}	11.53+-1.99	11.5+-1.98	11.64+-2.03	11.76+-2.09	11.64+-2.03
D^{mean}_{x55-60}	11.58+-2.01	11.55+-2	11.69+-2.05	11.81+-2.11	11.69+-2.04
T^{mean}_{x15}	11.19+-2.44	11.28+-2.47	10.89+-2.34	10.6+-2.24	10.89+-2.34
T^{max}_{x15}	21+-2.67	21.23+-2.62	20.39+-2.71	20.05+-2.74	20.39+-2.71
T^{min}_{x15}	2.42+-3.61	2.47+-3.56	2.16+-3.68	1.83+-3.69	2.17+-3.69
U^{mean}_{x15}	65.79+-12.09	64.93+-12.2	67.66+-11.39	68.19+-10.85	67.66+-11.4
P^{mean}_{x15}	7.31+-1.73	7.34+-1.73	7.18+-1.7	7.01+-1.64	7.19+-1.7
D^{sum}_{x15}	3401.96+-926.59	3407.87+-916.65	3412.69+-949.6	3454.65+-967.19	3411.68+-949.55
D^{max}_{x15}	10.99+-1.09	10.99+-1.07	11.02+-1.16	11.09+-1.2	11.02+-1.15
D^{min}_{x15}	10.56+-0.94	10.55+-0.92	10.6+-1	10.65+-1.05	10.6+-1
D^{mean}_{x15}	10.77+-1.02	10.76+-0.99	10.81+-1.08	10.86+-1.12	10.81+-1.08
T^{mean}_{x20}	11.12+-2.18	11.25+-2.17	10.72+-2.17	10.39+-2.1	10.72+-2.17
T^{max}_{x20}	21.63+-3.1	21.94+-3.04	20.85+-3.13	20.43+-3.14	20.86+-3.13
T^{min}_{x20}	1.31+-2.81	1.4+-2.83	1+-2.71	0.7+-2.63	1+-2.71
U^{mean}_{x20}	63.26+-12.32	62.33+-12.38	65.23+-11.63	65.72+-10.99	65.23+-11.64
P^{mean}_{x20}	7.38+-1.66	7.41+-1.66	7.26+-1.62	7.09+-1.57	7.26+-1.63
D^{sum}_{x20}	4478.3+-1233.11	4486.92+-1221.22	4490.47+-1260.32	4544.95+-1281.95	4489.14+-1260.27
D^{max}_{x20}	11.06+-1.17	11.06+-1.14	11.1+-1.23	11.17+-1.27	11.1+-1.23
D^{min}_{x20}	10.51+-0.96	10.5+-0.94	10.55+-1.02	10.6+-1.06	10.55+-1.02
D^{mean}_{x20}	10.78+-1.06	10.77+-1.04	10.82+-1.13	10.87+-1.17	10.82+-1.13
T^{mean}_{x25}	11.26+-1.78	11.37+-1.78	10.93+-1.73	10.66+-1.66	10.93+-1.73
T^{max}_{x25}	22.02+-2.91	22.28+-2.84	21.39+-3	21.08+-3.06	21.39+-3
T^{min}_{x25}	1.3+-2.8	1.39+-2.82	0.98+-2.7	0.69+-2.62	0.99+-2.7
U^{mean}_{x25}	63.11+-11.84	62.3+-11.89	64.81+-11.28	65.17+-10.76	64.81+-11.29
P^{mean}_{x25}	7.41+-1.7	7.43+-1.71	7.31+-1.66	7.15+-1.59	7.31+-1.66
D^{sum}_{x25}	5555.32+-1540.95	5566.78+-1527.57	5568.54+-1571.21	5635.25+-1596.37	5566.9+-1571.18
D^{max}_{x25}	11.14+-1.24	11.13+-1.21	11.18+-1.3	11.25+-1.34	11.18+-1.3
D^{min}_{x25}	10.46+-0.98	10.45+-0.96	10.51+-1.04	10.55+-1.08	10.5+-1.04
D^{mean}_{x25}	10.78+-1.11	10.78+-1.09	10.83+-1.17	10.89+-1.22	10.83+-1.17
T^{mean}_{x30}	11.36+-1.5	11.46+-1.49	11.07+-1.47	10.83+-1.41	11.07+-1.47
T^{max}_{x30}	22.14+-2.75	22.38+-2.69	21.55+-2.82	21.27+-2.87	21.55+-2.82
T^{min}_{x30}	1.07+-2.39	1.13+-2.38	0.82+-2.38	0.55+-2.33	0.82+-2.38

U^{mean}_{x30}	63.32+-10.76	62.59+-10.79	64.81+-10.29	65.07+-9.8	64.81+-10.3
P^{mean}_{x30}	7.41+-1.63	7.42+-1.64	7.31+-1.59	7.17+-1.54	7.31+-1.59
D^{sum}_{x30}	6579.82+-1921.32	6600.84+-1899.24	6574.24+-1976.28	6638.72+-2023.45	6572.52+-1975.98
D^{max}_{x30}	11.22+-1.31	11.21+-1.28	11.26+-1.37	11.34+-1.41	11.26+-1.37
D^{min}_{x30}	10.42+-1	10.4+-0.98	10.47+-1.06	10.51+-1.11	10.46+-1.06
D^{mean}_{x30}	10.8+-1.16	10.79+-1.14	10.84+-1.22	10.9+-1.27	10.84+-1.22
T^{mean}_{x35}	11.34+-1.26	11.41+-1.26	11.1+-1.24	10.91+-1.21	11.1+-1.25
T^{max}_{x35}	23.04+-3.02	23.22+-2.93	22.64+-3.18	22.51+-3.28	22.64+-3.18
T^{min}_{x35}	0.13+-1.83	0.16+-1.84	0.04+-1.83	-0.06+-1.86	0.04+-1.83
U^{mean}_{x35}	62.46+-11.23	61.71+-11.26	63.99+-10.74	64.26+-10.25	64+-10.75
P^{mean}_{x35}	7.43+-1.63	7.45+-1.64	7.33+-1.59	7.2+-1.55	7.34+-1.6
D^{sum}_{x35}	7592.17+-2338.19	7623.66+-2303.97	7564.77+-2427.21	7624.45+-2502.82	7563.01+-2426.52
D^{max}_{x35}	11.3+-1.37	11.3+-1.35	11.35+-1.43	11.42+-1.48	11.35+-1.43
D^{min}_{x35}	10.38+-1.02	10.37+-1	10.43+-1.08	10.48+-1.13	10.43+-1.08
D^{mean}_{x35}	10.81+-1.21	10.8+-1.19	10.86+-1.27	10.92+-1.32	10.86+-1.27
T^{mean}_{x40}	11.39+-1.34	11.45+-1.32	11.16+-1.36	10.98+-1.34	11.17+-1.36
T^{max}_{x40}	23.2+-2.89	23.38+-2.8	22.79+-3.08	22.63+-3.22	22.8+-3.08
T^{min}_{x40}	0.07+-1.79	0.09+-1.79	-0.01+-1.8	-0.1+-1.83	-0.01+-1.8
U^{mean}_{x40}	62.21+-10.99	61.48+-10.98	63.7+-10.63	63.97+-10.23	63.7+-10.64
P^{mean}_{x40}	7.44+-1.6	7.45+-1.61	7.35+-1.56	7.22+-1.51	7.35+-1.56
D^{sum}_{x40}	8618.14+-2773.58	8661.35+-2727.59	8565.8+-2895.28	8619.12+-2998.34	8564.02+-2894.23
D^{max}_{x40}	11.39+-1.44	11.38+-1.41	11.43+-1.5	11.51+-1.54	11.43+-1.5
D^{min}_{x40}	10.35+-1.04	10.34+-1.02	10.4+-1.1	10.45+-1.15	10.4+-1.1
D^{mean}_{x40}	10.83+-1.26	10.82+-1.23	10.88+-1.31	10.95+-1.36	10.88+-1.31
T^{mean}_{x45}	11.44+-1.38	11.49+-1.35	11.26+-1.44	11.11+-1.47	11.26+-1.44
T^{max}_{x45}	23.71+-3.67	23.82+-3.49	23.49+-4.12	23.47+-4.42	23.49+-4.12
T^{min}_{x45}	0.07+-1.79	0.09+-1.79	-0.01+-1.8	-0.1+-1.83	-0.01+-1.8
U^{mean}_{x45}	62.44+-11.08	61.72+-11.03	63.92+-10.84	64.19+-10.56	63.92+-10.85
P^{mean}_{x45}	7.39+-1.59	7.4+-1.61	7.3+-1.55	7.17+-1.5	7.3+-1.55
D^{sum}_{x45}	9574.56+-3283.73	9620.95+-3235.28	9517.78+-3417.81	9573.86+-3540.57	9515.9+-3416.49
D^{max}_{x45}	11.47+-1.5	11.46+-1.48	11.52+-1.56	11.6+-1.61	11.52+-1.56
D^{min}_{x45}	10.33+-1.06	10.31+-1.04	10.38+-1.11	10.42+-1.17	10.38+-1.11
D^{mean}_{x45}	10.85+-1.3	10.84+-1.28	10.91+-1.36	10.97+-1.41	10.91+-1.36
T^{mean}_{x50}	11.42+-1.52	11.44+-1.5	11.31+-1.57	11.19+-1.61	11.31+-1.57
T^{max}_{x50}	23.81+-3.61	23.91+-3.43	23.59+-4.08	23.54+-4.39	23.59+-4.07
T^{min}_{x50}	-0.34+-2.52	-0.37+-2.58	-0.31+-2.34	-0.34+-2.28	-0.31+-2.34
U^{mean}_{x50}	62.51+-11.1	61.8+-11.04	63.95+-10.92	64.22+-10.68	63.96+-10.92
P^{mean}_{x50}	7.4+-1.66	7.41+-1.67	7.3+-1.61	7.16+-1.56	7.3+-1.61
D^{sum}_{x50}	10537.76+-3810.65	10587.39+-3760.68	10476.3+-3954.56	10534.94+-4095.65	10474.32+-3952.98
D^{max}_{x50}	11.55+-1.56	11.55+-1.53	11.6+-1.61	11.68+-1.66	11.6+-1.61
D^{min}_{x50}	10.31+-1.07	10.3+-1.05	10.36+-1.13	10.4+-1.18	10.36+-1.13
D^{mean}_{x50}	10.88+-1.35	10.86+-1.33	10.93+-1.4	11+-1.46	10.93+-1.4
T^{mean}_{x55}	11.42+-1.65	11.44+-1.64	11.32+-1.69	11.22+-1.73	11.33+-1.69
T^{max}_{x55}	23.81+-3.61	23.91+-3.43	23.59+-4.08	23.54+-4.39	23.59+-4.07
T^{min}_{x55}	-0.34+-2.52	-0.37+-2.58	-0.31+-2.34	-0.34+-2.28	-0.31+-2.34
U^{mean}_{x55}	62.59+-10.96	61.84+-10.93	64.17+-10.71	64.56+-10.46	64.18+-10.71
P^{mean}_{x55}	7.4+-1.67	7.41+-1.68	7.29+-1.62	7.14+-1.57	7.29+-1.62
D^{sum}_{x55}	11500.98+-4343.96	11553.46+-4292.99	11435.66+-4496.32	11496.97+-4655	11433.58+-4494.49
D^{max}_{x55}	11.64+-1.61	11.63+-1.59	11.69+-1.66	11.76+-1.72	11.69+-1.66
D^{min}_{x55}	10.3+-1.08	10.28+-1.06	10.35+-1.14	10.39+-1.19	10.35+-1.14
D^{mean}_{x55}	10.9+-1.39	10.89+-1.37	10.96+-1.45	11.02+-1.5	10.96+-1.45
T^{mean}_{x60}	11.44+-1.84	11.45+-1.83	11.37+-1.85	11.29+-1.89	11.37+-1.85
T^{max}_{x60}	23.81+-3.61	23.91+-3.43	23.59+-4.08	23.54+-4.39	23.59+-4.07
T^{min}_{x60}	-0.41+-2.47	-0.44+-2.54	-0.36+-2.31	-0.38+-2.25	-0.36+-2.31
U^{mean}_{x60}	62.76+-10.94	62.02+-10.92	64.33+-10.64	64.71+-10.36	64.33+-10.65
P^{mean}_{x60}	7.4+-1.63	7.42+-1.64	7.3+-1.59	7.17+-1.54	7.31+-1.59
D^{sum}_{x60}	12362.91+-4877.95	12424.36+-4832.28	12273.64+-5023.41	12320.67+-5193.48	12271.75+-5021.39
D^{max}_{x60}	11.72+-1.66	11.71+-1.64	11.77+-1.71	11.84+-1.76	11.77+-1.71
D^{min}_{x60}	10.29+-1.09	10.28+-1.07	10.34+-1.14	10.38+-1.2	10.34+-1.14
D^{mean}_{x60}	10.93+-1.43	10.92+-1.41	10.99+-1.49	11.06+-1.54	10.99+-1.48
$T^{flowering10}$	18.72+-4.35	18.76+-4.43	17.15+-4.3	18.76+-4.16	18.19+-4.5
$U^{flowering10}$	44.53+-17.27	44.7+-18.74	49.86+-18.11	44.08+-16.18	47.48+-18.31
$P^{flowering10}$	9.34+-3.89	9.19+-3.48	8.87+-3.63	8.98+-3.33	8.86+-3.22
$D^{flowering10}$	13.31+-0.69	13.28+-0.72	13.22+-0.8	13.47+-0.7	13.31+-0.82

Table S3: Pearson's correlation coefficients between climatic factors and flowering time with corresponding p-values. D, T, U and P denote day length, temperature, relative humidity and precipitation, respectively. Superscripts `min`, `max` and `mean` denote minimum, maximum and mean values of a factor over a period of time defined in subscript where a number preceeding or succeeding `x` defines a day before or after sowing, respectively. Superscript `flowering10` a factor value at a day when 10% of plants flower.

Symbol	Adiyaman		Diyarbakir		Mardin		Sirnak		Urfa	
	corr	p.value	corr	p.value	corr	p.value	corr	p.value	corr	p.value
$T^{mean}_{sowing-flowering}$	-0.64	0	-0.68	0	-0.59	0	-0.6	0	-0.55	0
$T^{sum10}_{sowing-flowering}$	0.09	0.4305	0.06	0.263	0.15	0	0.23	0	0.06	0.2231
$T^{sum15}_{sowing-flowering}$	-0.4	3e-04	-0.43	0	-0.35	0	-0.28	0	-0.3	0
$T^{sum}_{sowing-flowering}$	0.21	0.0702	0.19	4e-04	0.27	0	0.34	0	0.15	0.0026
$T^{max}_{sowing-flowering}$	-0.16	0.1625	-0.29	0	-0.73	0	-0.15	0.0032	-0.59	0
$T^{min}_{sowing-flowering}$	0	0.9728	0.03	0.6293	0.27	0	0.3	0	0.2	1e-04
$U^{mean}_{sowing-flowering}$	0.9	0	0.9	0	0.93	0	0.92	0	0.86	0
$P^{mean}_{sowing-flowering}$	0.74	0	0.77	0	0.74	0	0.67	0	0.63	0
$P^{sum}_{sowing-flowering}$	0.97	0	0.97	0	0.98	0	0.98	0	0.97	0
$D^{sum}_{sowing-flowering}$	0.24	0.0342	0.17	0.0011	0.15	0	0.25	0	0.09	0.0578
$D^{max}_{sowing-flowering}$	-0.68	0	-0.64	0	-0.74	0	-0.75	0	-0.63	0
$D^{min}_{sowing-flowering}$	-0.43	1e-04	-0.46	0	-0.52	0	-0.49	0	-0.42	0
$D^{mean}_{sowing-flowering}$	-0.63	0	-0.62	0	-0.68	0	-0.68	0	-0.58	0
T^{sowing}	0.29	2e-04	0.4	0	0.22	0	0.13	1e-04	0.16	0
U^{sowing}	-0.15	0.0575	-0.18	0	-0.01	0.7746	0.07	0.0426	-0.03	0.3279
P^{sowing}	-0.31	1e-04	-0.18	0	-0.34	0	-0.42	0	-0.36	0
D^{sowing}	0.18	0.0209	0.05	0.138	0.01	0.6704	0.06	0.0828	0.02	0.5442
T^{mean}_{5x5}	0.55	0	0.62	0	0.53	0	0.51	0	0.48	0
T^{max}_{5x5}	0.59	0	0.64	0	0.58	0	0.58	0	0.54	0

T^{min}_{5x5}	0.27	4e-04	0.39	0	0.17	0	0.07	0.0327	0.12	7e-04
U^{mean}_{5x5}	0.12	0.1236	0.12	8e-04	0.15	0	0.18	0	0.12	4e-04
P^{mean}_{5x5}	-0.37	0	-0.23	0	-0.38	0	-0.46	0	-0.38	0
D^{sum}_{5x5}	0.3	1e-04	0.15	0	0.28	0	0.37	0	0.28	0
D^{max}_{5x5}	0.2	0.012	0.07	0.0565	0.03	0.2555	0.08	0.0289	0.04	0.2613
D^{min}_{5x5}	0.16	0.041	0.03	0.338	-0.01	0.6595	0.04	0.2396	0	0.9853
D^{mean}_{5x5}	0.18	0.0199	0.06	0.127	0.01	0.6576	0.06	0.0812	0.02	0.535
T^{mean}_{x10}	0.53	0	0.59	0	0.53	0	0.54	0	0.49	0
T^{max}_{x10}	0.61	0	0.65	0	0.6	0	0.61	0	0.56	0
T^{min}_{x10}	-0.03	0.6987	0.1	0.0045	-0.03	0.2403	-0.1	0.0056	-0.05	0.1346
U^{mean}_{x10}	0.1	0.1803	0.1	0.0069	0.19	0	0.23	0	0.17	0
P^{mean}_{x10}	-0.31	1e-04	-0.19	0	-0.29	0	-0.35	0	-0.31	0
D^{sum}_{x10}	0.3	1e-04	0.15	0	0.25	0	0.34	0	0.26	0
D^{max}_{x10}	0	0.9906	-0.11	0.0022	-0.14	0	-0.1	0.0026	-0.12	8e-04
D^{min}_{x10}	-0.05	0.5109	-0.15	0	-0.18	0	-0.15	0	-0.17	0
D^{mean}_{x10}	-0.02	0.7612	-0.13	3e-04	-0.16	0	-0.13	3e-04	-0.14	1e-04
T^{mean}_{10x}	0.6	0	0.64	0	0.59	0	0.6	0	0.56	0
T^{max}_{10x}	0.69	0	0.72	0	0.7	0	0.72	0	0.67	0
T^{min}_{10x}	0.29	1e-04	0.39	0	0.19	0	0.1	0.0054	0.15	0
U^{mean}_{10x}	0.18	0.0231	0.18	0	0.22	0	0.24	0	0.2	0
P^{mean}_{10x}	-0.26	8e-04	-0.13	3e-04	-0.32	0	-0.4	0	-0.34	0
D^{sum}_{10x}	0.46	0	0.37	0	0.61	0	0.66	0	0.6	0
D^{max}_{10x}	0.39	0	0.26	0	0.21	0	0.27	0	0.21	0
D^{min}_{10x}	0.33	0	0.2	0	0.14	0	0.21	0	0.15	0
D^{mean}_{10x}	0.37	0	0.24	0	0.18	0	0.24	0	0.18	0
T^{mean}_{x5}	0.52	0	0.58	0	0.5	0	0.48	0	0.45	0
T^{max}_{x5}	0.61	0	0.65	0	0.6	0	0.61	0	0.56	0
T^{min}_{x5}	0.03	0.663	0.17	0	-0.01	0.5362	-0.1	0.0047	-0.07	0.0471
U^{mean}_{x5}	0.04	0.629	0.02	0.5006	0.08	9e-04	0.13	3e-04	0.06	0.1107
P^{mean}_{x5}	-0.32	0	-0.19	0	-0.3	0	-0.38	0	-0.32	0
D^{sum}_{x5}	0.34	0	0.19	0	0.29	0	0.37	0	0.29	0
D^{max}_{x5}	0.09	0.2699	-0.03	0.3775	-0.07	0.0063	-0.03	0.4629	-0.05	0.1421
D^{min}_{x5}	0.06	0.4077	-0.05	0.1571	-0.09	3e-04	-0.05	0.1898	-0.07	0.0364
D^{mean}_{x5}	0.08	0.3181	-0.04	0.2797	-0.08	0.0019	-0.03	0.3255	-0.06	0.0819
T^{mean}_{x5-15}	0.14	0.0823	0.18	0	0.14	0	0.17	0	0.11	0.0014
T^{max}_{x5-15}	0.06	0.4635	0.01	0.8631	0.12	0	0.21	0	0.12	9e-04
T^{min}_{x5-15}	-0.15	0.0534	-0.06	0.1092	-0.13	0	-0.16	0	-0.17	0
U^{mean}_{x5-15}	-0.05	0.5519	-0.03	0.4331	0.03	0.1952	0.01	0.6933	0.01	0.8647
P^{mean}_{x5-15}	-0.21	0.0066	-0.11	0.002	-0.2	0	-0.23	0	-0.22	0
D^{sum}_{x5-15}	0.2	0.0083	0.06	0.1297	0.17	0	0.26	0	0.18	0
D^{max}_{x5-15}	-0.18	0.0244	-0.27	0	-0.27	0	-0.26	0	-0.25	0
D^{min}_{x5-15}	-0.24	0.0022	-0.31	0	-0.33	0	-0.31	0	-0.31	0
D^{mean}_{x5-15}	-0.21	0.0076	-0.29	0	-0.3	0	-0.29	0	-0.28	0
T^{mean}_{x5-10}	0.54	0	0.59	0	0.57	0	0.61	0	0.54	0
T^{max}_{x5-10}	0.49	0	0.51	0	0.48	0	0.54	0	0.47	0
T^{min}_{x5-10}	0.51	0	0.55	0	0.57	0	0.58	0	0.56	0
U^{mean}_{x5-10}	0.14	0.0784	0.14	1e-04	0.25	0	0.27	0	0.22	0
P^{mean}_{x5-10}	-0.27	5e-04	-0.18	0	-0.24	0	-0.26	0	-0.26	0
D^{sum}_{x5-10}	0.3	1e-04	0.15	0	0.24	0	0.33	0	0.24	0
D^{max}_{x5-10}	-0.1	0.183	-0.2	0	-0.22	0	-0.2	0	-0.2	0
D^{min}_{x5-10}	-0.13	0.0895	-0.23	0	-0.24	0	-0.22	0	-0.23	0
D^{mean}_{x5-10}	-0.12	0.1247	-0.22	0	-0.23	0	-0.21	0	-0.21	0
T^{mean}_{x10-15}	-0.27	5e-04	-0.26	0	-0.29	0	-0.26	0	-0.31	0
T^{max}_{x10-15}	-0.33	0	-0.37	0	-0.29	0	-0.22	0	-0.29	0
T^{min}_{x10-15}	-0.23	0.0032	-0.17	0	-0.26	0	-0.27	0	-0.29	0
U^{mean}_{x10-15}	-0.18	0.0222	-0.15	0	-0.12	0	-0.15	0	-0.14	1e-04
P^{mean}_{x10-15}	-0.09	0.2442	0.01	0.7264	-0.11	0	-0.14	0	-0.11	0.0014
D^{sum}_{x10-15}	0.1	0.2055	-0.05	0.2066	0.1	0	0.19	0	0.11	0.0027
D^{max}_{x10-15}	-0.27	5e-04	-0.34	0	-0.35	0	-0.34	0	-0.32	0
D^{min}_{x10-15}	-0.3	1e-04	-0.37	0	-0.37	0	-0.37	0	-0.35	0
D^{mean}_{x10-15}	-0.28	2e-04	-0.36	0	-0.36	0	-0.35	0	-0.34	0
T^{mean}_{x15-20}	-0.21	0.0056	-0.22	0	-0.08	0.0014	-0.02	0.5305	-0.09	0.0083
T^{max}_{x15-20}	-0.06	0.4331	-0.12	0.0014	0	0.9209	0.07	0.0579	-0.01	0.6771
T^{min}_{x15-20}	-0.41	0	-0.39	0	-0.32	0	-0.31	0	-0.32	0
U^{mean}_{x15-20}	-0.19	0.0156	-0.16	0	-0.17	0	-0.2	0	-0.18	0
P^{mean}_{x15-20}	-0.07	0.4055	0.03	0.3663	-0.06	0.0102	-0.09	0.0135	-0.07	0.055
D^{sum}_{x15-20}	0.06	0.4728	-0.09	0.0171	0.05	0.0297	0.14	1e-04	0.06	0.0885
D^{max}_{x15-20}	-0.4	0	-0.46	0	-0.45	0	-0.46	0	-0.43	0
D^{min}_{x15-20}	-0.43	0	-0.48	0	-0.48	0	-0.48	0	-0.45	0
D^{mean}_{x15-20}	-0.41	0	-0.47	0	-0.47	0	-0.47	0	-0.44	0
T^{mean}_{x20-25}	-0.13	0.0982	-0.18	0	-0.19	0	-0.14	0	-0.15	0
T^{max}_{x20-25}	-0.04	0.6383	-0.08	0.0299	-0.13	0	-0.1	0.0046	-0.15	0
T^{min}_{x20-25}	-0.05	0.4999	-0.06	0.0862	0.08	0.0015	0.1	0.0042	0.1	0.0033
U^{mean}_{x20-25}	-0.23	0.0024	-0.22	0	-0.08	0.0012	-0.07	0.0415	-0.1	0.0039
P^{mean}_{x20-25}	-0.16	0.0343	-0.04	0.2933	-0.24	0	-0.32	0	-0.26	0
D^{sum}_{x20-25}	-0.02	0.8468	-0.15	0	-0.01	0.6127	0.07	0.0425	0	0.9161
D^{max}_{x20-25}	-0.5	0	-0.55	0	-0.54	0	-0.55	0	-0.51	0
D^{min}_{x20-25}	-0.53	0	-0.56	0	-0.56	0	-0.57	0	-0.53	0
D^{mean}_{x20-25}	-0.52	0	-0.55	0	-0.55	0	-0.56	0	-0.52	0
T^{mean}_{x25-30}	-0.67	0	-0.71	0	-0.66	0	-0.64	0	-0.64	0
T^{max}_{x25-30}	-0.51	0	-0.57	0	-0.52	0	-0.49	0	-0.5	0
T^{min}_{x25-30}	-0.58	0	-0.64	0	-0.49	0	-0.43	0	-0.45	0
U^{mean}_{x25-30}	0.49	0	0.52	0	0.53	0	0.55	0	0.5	0
P^{mean}_{x25-30}	-0.3	1e-04	-0.17	0	-0.34	0	-0.44	0	-0.34	0
D^{sum}_{x25-30}	-0.05	0.518	-0.17	0	-0.05	0.0303	0.01	0.6841	-0.05	0.1302
D^{max}_{x25-30}	-0.58	0	-0.61	0	-0.61	0	-0.62	0	-0.58	0
D^{min}_{x25-30}	-0.6	0	-0.63	0	-0.62	0	-0.64	0	-0.59	0
D^{mean}_{x25-30}	-0.59	0	-0.62	0	-0.61	0	-0.63	0	-0.58	0
T^{mean}_{x30-35}	-0.92	0	-0.91	0	-0.93	0	-0.95	0	-0.91	0
T^{max}_{x30-35}	-0.81	0	-0.81	0	-0.85	0	-0.89	0	-0.83	0
T^{min}_{x30-35}	-0.68	0	-0.7	0	-0.72	0	-0.77	0	-0.67	0
U^{mean}_{x30-35}	0.2	0.0149	0.16	0	0.39	0	0.51	0	0.36	0

P^{mean}_{x30-35}	-0.29	4e-04	-0.15	1e-04	-0.39	0	-0.5	0	-0.4	0
D^{sum}_{x30-35}	-0.08	0.3278	-0.21	0	-0.09	8e-04	-0.02	0.644	-0.07	0.0764
D^{max}_{x30-35}	-0.67	0	-0.68	0	-0.69	0	-0.73	0	-0.65	0
D^{min}_{x30-35}	-0.68	0	-0.69	0	-0.7	0	-0.74	0	-0.66	0
D^{mean}_{x30-35}	-0.68	0	-0.69	0	-0.7	0	-0.73	0	-0.66	0
T^{mean}_{x35-40}	-0.83	0	-0.83	0	-0.86	0	-0.88	0	-0.88	0
T^{max}_{x35-40}	-0.79	0	-0.78	0	-0.83	0	-0.84	0	-0.86	0
T^{min}_{x35-40}	-0.88	0	-0.87	0	-0.91	0	-0.93	0	-0.9	0
U^{mean}_{x35-40}	0.35	0	0.35	0	0.49	0	0.55	0	0.48	0
P^{mean}_{x35-40}	-0.16	0.0596	-0.05	0.2275	-0.2	0	-0.25	0	-0.21	0
D^{sum}_{x35-40}	-0.16	0.0558	-0.28	0	-0.15	0	-0.09	0.0226	-0.13	7e-04
D^{max}_{x35-40}	-0.72	0	-0.73	0	-0.73	0	-0.77	0	-0.7	0
D^{min}_{x35-40}	-0.72	0	-0.73	0	-0.74	0	-0.78	0	-0.7	0
D^{mean}_{x35-40}	-0.72	0	-0.73	0	-0.74	0	-0.78	0	-0.7	0
T^{mean}_{x40-45}	-0.47	0	-0.51	0	-0.54	0	-0.54	0	-0.51	0
T^{max}_{x40-45}	-0.44	0	-0.48	0	-0.51	0	-0.51	0	-0.51	0
T^{min}_{x40-45}	-0.41	0	-0.49	0	-0.41	0	-0.38	0	-0.34	0
U^{mean}_{x40-45}	0.66	0	0.66	0	0.75	0	0.79	0	0.73	0
P^{mean}_{x40-45}	-0.26	0.0017	-0.19	0	-0.36	0	-0.43	0	-0.37	0
D^{sum}_{x40-45}	-0.14	0.0893	-0.24	0	-0.15	0	-0.1	0.0084	-0.14	2e-04
D^{max}_{x40-45}	-0.75	0	-0.76	0	-0.77	0	-0.8	0	-0.73	0
D^{min}_{x40-45}	-0.76	0	-0.76	0	-0.77	0	-0.81	0	-0.73	0
D^{mean}_{x40-45}	-0.76	0	-0.76	0	-0.77	0	-0.81	0	-0.73	0
T^{mean}_{x45-50}	-0.67	0	-0.69	0	-0.69	0	-0.69	0	-0.66	0
T^{max}_{x45-50}	-0.74	0	-0.75	0	-0.77	0	-0.77	0	-0.74	0
T^{min}_{x45-50}	-0.14	0.1191	-0.24	0	-0.14	0	-0.06	0.1487	-0.04	0.3808
U^{mean}_{x45-50}	0.64	0	0.64	0	0.72	0	0.78	0	0.69	0
P^{mean}_{x45-50}	-0.28	0.0019	-0.18	0	-0.28	0	-0.32	0	-0.31	0
D^{sum}_{x45-50}	-0.25	0.0054	-0.36	0	-0.24	0	-0.18	0	-0.2	0
D^{max}_{x45-50}	-0.82	0	-0.82	0	-0.82	0	-0.86	0	-0.78	0
D^{min}_{x45-50}	-0.82	0	-0.81	0	-0.82	0	-0.85	0	-0.78	0
D^{mean}_{x45-50}	-0.82	0	-0.81	0	-0.82	0	-0.86	0	-0.78	0
T^{mean}_{x50-55}	-0.93	0	-0.94	0	-0.95	0	-0.96	0	-0.96	0
T^{max}_{x50-55}	-0.92	0	-0.93	0	-0.92	0	-0.91	0	-0.91	0
T^{min}_{x50-55}	-0.7	0	-0.74	0	-0.73	0	-0.76	0	-0.68	0
U^{mean}_{x50-55}	0.46	0	0.41	0	0.51	0	0.57	0	0.51	0
P^{mean}_{x50-55}	-0.25	0.0058	-0.12	0.0053	-0.23	0	-0.31	0	-0.26	0
D^{sum}_{x50-55}	-0.27	0.0026	-0.37	0	-0.26	0	-0.21	0	-0.23	0
D^{max}_{x50-55}	-0.84	0	-0.84	0	-0.84	0	-0.88	0	-0.81	0
D^{min}_{x50-55}	-0.84	0	-0.83	0	-0.84	0	-0.87	0	-0.8	0
D^{mean}_{x50-55}	-0.84	0	-0.83	0	-0.84	0	-0.87	0	-0.8	0
T^{mean}_{x55-60}	-0.94	0	-0.94	0	-0.96	0	-0.96	0	-0.96	0
T^{max}_{x55-60}	-0.97	0	-0.96	0	-0.97	0	-0.98	0	-0.96	0
T^{min}_{x55-60}	-0.86	0	-0.87	0	-0.88	0	-0.89	0	-0.87	0
U^{mean}_{x55-60}	0.72	0	0.7	0	0.73	0	0.79	0	0.7	0
P^{mean}_{x55-60}	-0.36	0	-0.21	0	-0.31	0	-0.41	0	-0.31	0
D^{sum}_{x55-60}	-0.72	0	-0.73	0	-0.76	0	-0.79	0	-0.77	0
D^{max}_{x55-60}	-0.86	0	-0.86	0	-0.86	0	-0.89	0	-0.83	0
D^{min}_{x55-60}	-0.85	0	-0.85	0	-0.85	0	-0.88	0	-0.82	0
D^{mean}_{x55-60}	-0.85	0	-0.85	0	-0.86	0	-0.89	0	-0.82	0
T^{mean}_{x15}	0.28	3e-04	0.34	0	0.27	0	0.28	0	0.23	0
T^{max}_{x15}	0.28	3e-04	0.26	0	0.3	0	0.35	0	0.27	0
T^{min}_{x15}	-0.13	0.1008	-0.03	0.3785	-0.12	0	-0.15	0	-0.15	0
U^{mean}_{x15}	-0.02	0.8365	-0.01	0.7774	0.05	0.0265	0.06	0.0807	0.03	0.4241
P^{mean}_{x15}	-0.26	9e-04	-0.14	1e-04	-0.26	0	-0.31	0	-0.27	0
D^{sum}_{x15}	0.24	0.0023	0.09	0.0182	0.2	0	0.29	0	0.21	0
D^{max}_{x15}	-0.08	0.3068	-0.18	0	-0.2	0	-0.17	0	-0.18	0
D^{min}_{x15}	-0.17	0.0333	-0.25	0	-0.27	0	-0.25	0	-0.25	0
D^{mean}_{x15}	-0.12	0.1249	-0.22	0	-0.23	0	-0.21	0	-0.21	0
T^{mean}_{x20}	0.16	0.0441	0.21	0	0.19	0	0.21	0	0.15	0
T^{max}_{x20}	0.13	0.0958	0.09	0.0165	0.19	0	0.25	0	0.16	0
T^{min}_{x20}	-0.18	0.0228	-0.09	0.0147	-0.17	0	-0.2	0	-0.19	0
U^{mean}_{x20}	-0.05	0.4865	-0.04	0.2542	0	0.851	0.01	0.8497	-0.02	0.5933
P^{mean}_{x20}	-0.22	0.0038	-0.11	0.0031	-0.23	0	-0.28	0	-0.24	0
D^{sum}_{x20}	0.19	0.0139	0.04	0.2516	0.17	0	0.26	0	0.17	0
D^{max}_{x20}	-0.15	0.0544	-0.24	0	-0.25	0	-0.24	0	-0.23	0
D^{min}_{x20}	-0.27	4e-04	-0.34	0	-0.36	0	-0.34	0	-0.33	0
D^{mean}_{x20}	-0.21	0.0076	-0.29	0	-0.3	0	-0.29	0	-0.28	0
T^{mean}_{x25}	0.1	0.1986	0.15	1e-04	0.12	0	0.15	0	0.1	0.0067
T^{max}_{x25}	0.03	0.6567	-0.02	0.5345	0.04	0.0888	0.1	0.0054	0.02	0.621
T^{min}_{x25}	-0.18	0.0239	-0.09	0.016	-0.17	0	-0.2	0	-0.18	0
U^{mean}_{x25}	-0.08	0.323	-0.07	0.0649	0.01	0.7325	0.02	0.6224	-0.02	0.6528
P^{mean}_{x25}	-0.23	0.0034	-0.1	0.0046	-0.25	0	-0.32	0	-0.26	0
D^{sum}_{x25}	0.15	0.0567	0	0.9824	0.13	0	0.22	0	0.14	1e-04
D^{max}_{x25}	-0.21	0.0063	-0.3	0	-0.3	0	-0.29	0	-0.28	0
D^{min}_{x25}	-0.37	0	-0.42	0	-0.43	0	-0.43	0	-0.41	0
D^{mean}_{x25}	-0.29	2e-04	-0.36	0	-0.36	0	-0.36	0	-0.34	0
T^{mean}_{x30}	-0.03	0.6756	0	0.971	-0.01	0.7756	0.02	0.4904	-0.03	0.4559
T^{max}_{x30}	-0.02	0.8059	-0.07	0.0473	-0.03	0.2273	0.01	0.7462	-0.05	0.1272
T^{min}_{x30}	-0.28	3e-04	-0.18	0	-0.23	0	-0.26	0	-0.25	0
U^{mean}_{x30}	0.01	0.872	0.02	0.5275	0.11	0	0.14	1e-04	0.08	0.0224
P^{mean}_{x30}	-0.23	0.0026	-0.11	0.0039	-0.26	0	-0.33	0	-0.27	0
D^{sum}_{x30}	0.11	0.1791	-0.04	0.3103	0.09	2e-04	0.17	0	0.09	0.0082
D^{max}_{x30}	-0.27	5e-04	-0.35	0	-0.35	0	-0.34	0	-0.32	0
D^{min}_{x30}	-0.45	0	-0.48	0	-0.49	0	-0.5	0	-0.47	0
D^{mean}_{x30}	-0.36	0	-0.42	0	-0.42	0	-0.42	0	-0.39	0
T^{mean}_{x35}	-0.3	1e-04	-0.27	0	-0.33	0	-0.33	0	-0.35	0
T^{max}_{x35}	-0.28	3e-04	-0.32	0	-0.36	0	-0.37	0	-0.37	0
T^{min}_{x35}	-0.49	0	-0.43	0	-0.38	0	-0.4	0	-0.37	0
U^{mean}_{x35}	0.03	0.6718	0.03	0.3589	0.15	0	0.2	0	0.13	4e-04
P^{mean}_{x35}	-0.24	0.002	-0.11	0.0029	-0.27	0	-0.35	0	-0.28	0
D^{sum}_{x35}	0.07	0.3473	-0.06	0.084	0.06	0.0169	0.14	1e-04	0.06	0.086
D^{max}_{x35}	-0.32	0	-0.4	0	-0.39	0	-0.39	0	-0.36	0

D^{min}_{x35}	-0.52	0	-0.54	0	-0.55	0	-0.56	0	-0.52	0
D^{mean}_{x35}	-0.42	0	-0.47	0	-0.47	0	-0.47	0	-0.44	0
T^{mean}_{x40}	-0.48	0	-0.46	0	-0.52	0	-0.53	0	-0.54	0
T^{max}_{x40}	-0.31	1e-04	-0.35	0	-0.39	0	-0.39	0	-0.4	0
T^{min}_{x40}	-0.51	0	-0.44	0	-0.39	0	-0.4	0	-0.38	0
U^{mean}_{x40}	0.07	0.3815	0.07	0.052	0.19	0	0.24	0	0.17	0
P^{mean}_{x40}	-0.23	0.0029	-0.1	0.0054	-0.27	0	-0.34	0	-0.28	0
D^{sum}_{x40}	0.04	0.5924	-0.09	0.0136	0.03	0.2276	0.1	0.0025	0.03	0.378
D^{max}_{x40}	-0.37	0	-0.44	0	-0.43	0	-0.43	0	-0.4	0
D^{min}_{x40}	-0.57	0	-0.58	0	-0.59	0	-0.6	0	-0.56	0
D^{mean}_{x40}	-0.47	0	-0.52	0	-0.51	0	-0.52	0	-0.48	0
T^{mean}_{x45}	-0.56	0	-0.56	0	-0.61	0	-0.6	0	-0.61	0
T^{max}_{x45}	-0.34	0	-0.4	0	-0.43	0	-0.43	0	-0.43	0
T^{min}_{x45}	-0.51	0	-0.44	0	-0.39	0	-0.4	0	-0.38	0
U^{mean}_{x45}	0.13	0.0938	0.14	2e-04	0.26	0	0.31	0	0.24	0
P^{mean}_{x45}	-0.23	0.0027	-0.1	0.0041	-0.28	0	-0.35	0	-0.28	0
D^{sum}_{x45}	0.01	0.8682	-0.11	0.0016	0	0.8841	0.08	0.0275	0	0.9105
D^{max}_{x45}	-0.41	0	-0.47	0	-0.46	0	-0.46	0	-0.43	0
D^{min}_{x45}	-0.61	0	-0.62	0	-0.62	0	-0.64	0	-0.59	0
D^{mean}_{x45}	-0.52	0	-0.56	0	-0.55	0	-0.56	0	-0.52	0
T^{mean}_{x50}	-0.64	0	-0.65	0	-0.69	0	-0.68	0	-0.69	0
T^{max}_{x50}	-0.36	0	-0.41	0	-0.44	0	-0.43	0	-0.45	0
T^{min}_{x50}	-0.55	0	-0.51	0	-0.44	0	-0.44	0	-0.42	0
U^{mean}_{x50}	0.18	0.0183	0.19	0	0.32	0	0.37	0	0.29	0
P^{mean}_{x50}	-0.24	0.0019	-0.11	0.0016	-0.28	0	-0.35	0	-0.29	0
D^{sum}_{x50}	-0.01	0.8878	-0.13	2e-04	-0.02	0.4518	0.05	0.129	-0.02	0.5977
D^{max}_{x50}	-0.45	0	-0.51	0	-0.49	0	-0.49	0	-0.47	0
D^{min}_{x50}	-0.64	0	-0.64	0	-0.65	0	-0.66	0	-0.62	0
D^{mean}_{x50}	-0.56	0	-0.59	0	-0.58	0	-0.59	0	-0.55	0
T^{mean}_{x55}	-0.72	0	-0.73	0	-0.77	0	-0.76	0	-0.76	0
T^{max}_{x55}	-0.36	0	-0.41	0	-0.44	0	-0.43	0	-0.45	0
T^{min}_{x55}	-0.55	0	-0.51	0	-0.44	0	-0.44	0	-0.42	0
U^{mean}_{x55}	0.2	0.0083	0.2	0	0.33	0	0.38	0	0.31	0
P^{mean}_{x55}	-0.24	0.0021	-0.11	0.0021	-0.27	0	-0.34	0	-0.28	0
D^{sum}_{x55}	-0.03	0.7077	-0.15	0	-0.04	0.1429	0.03	0.3323	-0.04	0.3017
D^{max}_{x55}	-0.48	0	-0.54	0	-0.52	0	-0.52	0	-0.5	0
D^{min}_{x55}	-0.66	0	-0.66	0	-0.66	0	-0.68	0	-0.63	0
D^{mean}_{x55}	-0.59	0	-0.62	0	-0.61	0	-0.62	0	-0.58	0
T^{mean}_{x60}	-0.79	0	-0.8	0	-0.82	0	-0.82	0	-0.82	0
T^{max}_{x60}	-0.36	0	-0.41	0	-0.44	0	-0.43	0	-0.45	0
T^{min}_{x60}	-0.57	0	-0.55	0	-0.46	0	-0.46	0	-0.44	0
U^{mean}_{x60}	0.24	0.0015	0.25	0	0.37	0	0.42	0	0.34	0
P^{mean}_{x60}	-0.26	9e-04	-0.13	4e-04	-0.29	0	-0.37	0	-0.3	0
D^{sum}_{x60}	-0.09	0.2275	-0.21	0	-0.11	0	-0.04	0.2182	-0.11	0.0018
D^{max}_{x60}	-0.51	0	-0.57	0	-0.54	0	-0.55	0	-0.52	0
D^{min}_{x60}	-0.67	0	-0.67	0	-0.67	0	-0.69	0	-0.64	0
D^{mean}_{x60}	-0.61	0	-0.64	0	-0.63	0	-0.64	0	-0.6	0
$T^{flowering10}$	-0.33	0	-0.41	0	-0.48	0	-0.46	0	-0.34	0
$U^{flowering10}$	0.14	0.071	0.19	0	0.13	0	0.19	0	0.11	0.0024
$P^{flowering10}$	-0.39	0	-0.34	0	-0.46	0	-0.44	0	-0.4	0
$D^{flowering10}$	0.2	0.0101	0.1	0.0065	0.13	0	0.16	0	0.17	0

Table S4: Reference allele frequency at six polymorphic sites associated with flowering time, as well as mean annual precipitation and mean annual temperature for different collection sites. Collection site is the site at which the genotypes were collected. The population names are derived from their collection site.

Collection site	Mean annual prec	Mean annual temp	SNP1	SNP2	SNP3	SNP4	SNP5	SNP6
Baristepe1	684.0	16.46	1.00	0.00	1.00	1.00	1.00	0.81
Baristepe2	684.0	16.46	0.60	0.40	0.60	1.00	1.00	0.80
Baristepe3	684.0	16.46	0.91	0.69	0.84	1.00	1.00	0.83
Beslever	687.5	16.03	1.00	0.63	0.63	0.83	1.00	0.75
Cudi	810.0	14.37	0.63	0.45	0.00	1.00	1.00	1.00
Cudi2	810.0	14.37	0.95	0.00	0.05	1.00	1.00	1.00
Dereici	704.0	15.89	0.91	0.95	0.80	0.95	0.95	0.82
Egill	720.5	14.84	0.55	0.35	0.50	0.60	0.65	0.70
Kalkan	674.0	15.15	0.88	1.00	1.00	0.88	0.86	0.71
Kayatepe	742.0	15.56	0.93	1.00	1.00	1.00	1.00	1.00
Kesentas	632.0	15.31	0.00	0.46	0.00	0.20	0.42	0.09
Oyali	552.5	14.31	0.36	0.00	0.04	0.12	0.29	0.50
Sarikaya	686.5	16.03	0.83	0.70	0.61	1.00	1.00	0.89
Savur	673.0	16.10	0.25	1.00	1.00	1.00	1.00	0.75
Sirnak	848.0	11.41	1.00	0.07	0.98	1.00	0.97	0.96

Table S5: List of SNPs associated with flowering time.

SNP	Chromosome	Position	Reference allele	Minor allele
1	1	5336589	G	T
2	1	13049018	G	A
3	2	25818800	T	C
4	4	19706556	T	C
5	4	19706954	A	C
6	5	40157119	G	A

Table S6: Number of times the reference allele for a SNP associated with flowering time is present in plant genotypes.

Genotype	SNP1	SNP2	SNP3	SNP4	SNP5	SNP6
Bari1_063	2	0	2	2	2	2
Bari1_064	2	0	2	2	2	2
Bari1_068	2	0	2	2	2	2
Bari1_069	2	0	2	2	2	1
Bari1_091	2	0	2	2	2	2
Bari1_092	2	0	2	2	2	2
Bari1_093	2	0	2	2	2	2
Bari2_062	2	1	2	2	2	2
Bari2_064	2	1	0	2	2	2
Bari2_067	2	2	0	2	2	2
Bari2_074	0	0	2	2	2	0
Bari3_074	2	1	2	2	2	2
Bari3_079	2	2	2	2	2	2
Bari3_091	2	1	2	2	2	2
Bari3_100	1	1	2	2	2	1
Bari3_101	1	1	1	2	2	1
Bari3_110	2	2	0	2	2	0
Besev_062	2	2	0	2	2	2
Besev_065	2	0	2	2	2	2
Besev_066	2	2	2	2	2	2
Besev_074	2	0	0	0	2	2
Besev_075	2	0	0	2	2	2
Besev_079	2	2	2	2	2	2
Besev_083	2	2	2	2	2	0
Cudi_101	2	0	0	2	2	2
Cudi_104	0	0	0	2	2	2
Cudi_124	0	0	0	2	2	2
Cudi_127	0	0	0	2	2	2
Cudi_128	2	2	0	2	2	2
Cudi_151	2	2	0	2	2	2
CudiA_152	2	2	0	2	2	2
Cudi_155	2	2	1	2	2	2
Cudi2_004	2	0	0	2	2	2
Cudi2_005	2	0	0	2	2	2
Cudi2_006	2	0	0	2	2	2
CudiB_008	2	0	0	2	2	2
Cudi2_009	2	1	0	2	2	2
Cudi2_018	2	0	0	2	2	2
Cudi2_019	1	0	0	2	2	2
Cudi2_023	2	0	0	2	2	2
Derei_062	0	2	0	2	2	0
Derei_065	2	2	2	2	2	2
Derei_066	2	2	2	2	2	2
Derei_069	2	1	1	2	2	2
Derei_070	2	2	2	2	2	2
Derei_072	2	2	2	2	2	2
Derei_073	2	2	2	1	1	1
Derei_074	2	2	0	2	2	1
Derei_075	2	2	2	2	2	2
Egill_063	0	0	0	0	0	2
Egill_065	0	0	1	0	0	2
Egill_066	0	0	1	0	0	2
Egill_073	2	2	2	2	2	2
Egill_074	2	2	2	2	2	2
Egill_075	2	1	1	2	2	2
Kalka_061	2	2	2	2	2	2
Kalka_064	2	2	2	2	2	2
Kalka_067	0	1	2	0	0	0
Kalka_070	2	2	2	2	2	2
Kalka_074	2	2	2	2	2	2
Kayat_061	2	2	2	2	2	2
Kayat_063	2	2	2	2	2	2
Kayat_064	1	2	2	2	2	2
Kayat_066	2	2	2	2	2	2
Kayat_070	2	2	2	2	2	2
Kayat_077	2	2	2	2	2	2
Kayat_080	2	2	2	2	2	2
Kesen_065	2	1	0	2	2	0
Kesen_071	0	0	0	0	0	1
Kesen_074	2	0	0	0	0	0
Kesen_075	0	0	0	0	0	0
Kesen_077	0	0	0	0	1	1
Kesen_104	0	2	0	2	2	0
Oyali_073	2	0	0	0	0	2
Oyali_076	2	0	0	0	1	0
Oyali_084	0	0	0	0	0	0
Oyali_100	0	0	0	0	0	0
Oyali_107	2	0	0	2	2	2
Sarik_064	2	2	0	2	2	0
Sarik_065	2	2	1	2	2	2
Sarik_066	0	0	0	2	2	2
Sarik_067	2	2	2	2	2	2
Sarik_073	2	2	2	2	2	2
Sarik_074	2	2	0	2	2	2
Sarik_077	1	2	2	2	2	2
Sarik_078	2	2	2	2	2	2
Sarik_080	2	0	1	2	2	2
Savur_063	1	2	2	2	2	2
Sirna_060	2	0	2	2	2	2
Sirna_064	2	1	2	2	2	1

Figure S1: Correlations of mean annual temperature with allele frequency of the 6 GWAS SNPs calculated for 15 populations of the wild chickpeas (shown for completeness). There are no significant correlations with the allele frequency of the snps with mean annual temperature meaning that the alleles do not directly correspond to pathways responsive in flowering time and the ambient temperature.

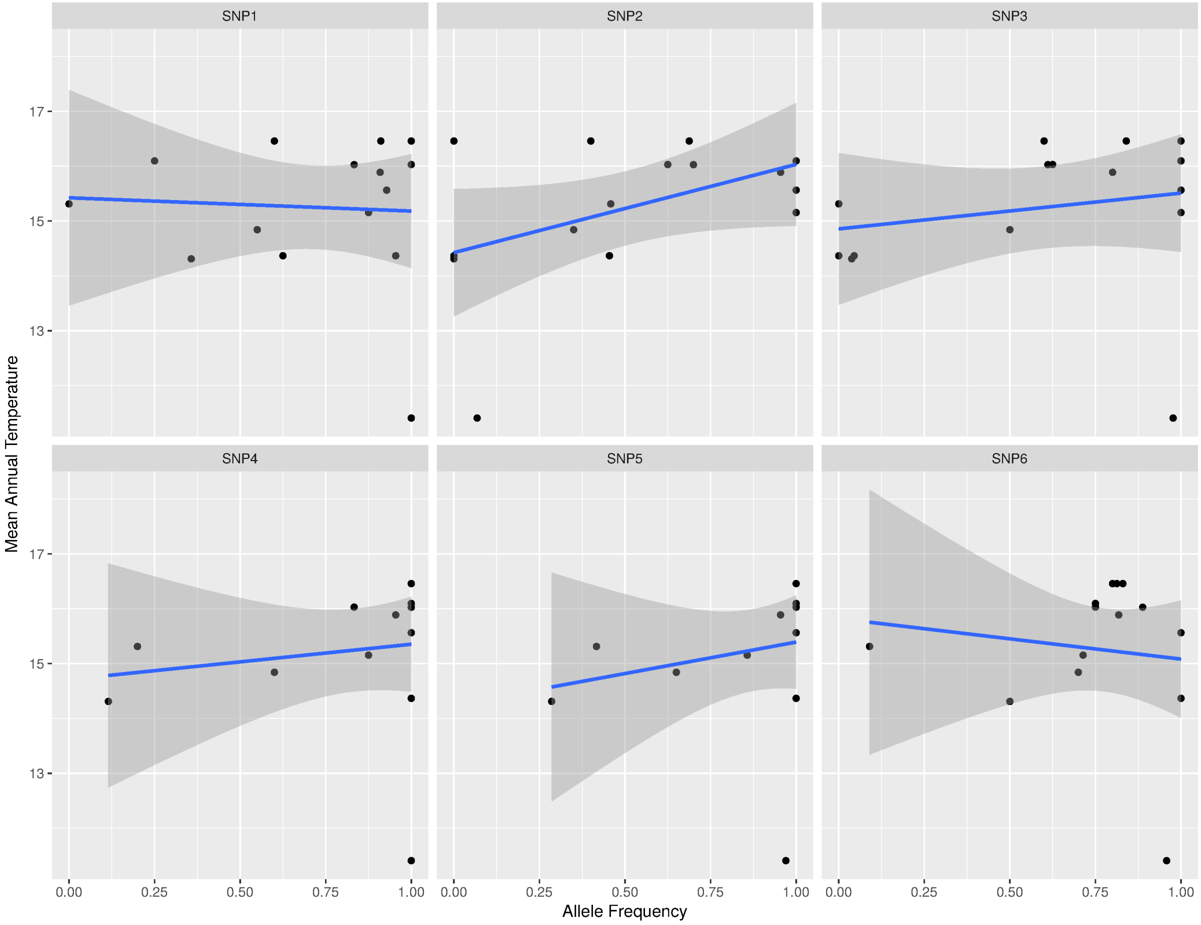

S2 Grammatical evolution method

The method was developed recently to recover the analytic form of a function from known values [O'Neill and Ryan, 2001, Noorian et al., 2016].

Formal definition of a context-free grammar (CFG):

A *formal grammar* where every production rule, formalized by the pair (n, V), is in form of $n \to V$.

The CFG is defined by the 4-tuple $(\mathcal{T}, \mathcal{N}, \mathcal{R}, \mathcal{S})$, where

- $\mathcal{T}$ is the finite set of terminal symbols,
- $\mathcal{N}$ is the finite set of non-terminal symbols,
- $\mathcal{R}$ is the production rule set,
- $\mathcal{S} \in \mathcal{N}$ is the start symbol.

A production rule $n \to V$ is realized by replacing the non-terminal symbol $n \in \mathcal{N}$ with the symbol $v \in V$, where $V \in (\mathcal{T} \cup \mathcal{N})^*$ is a sequence of terminal and/or non-terminal symbols [Aho et al., 2006].

Using the following grammar (see Tab. S7):

Table S7: Example grammar.

<expr>	::=	(<expr>)<op>(<expr>) | <coef>*<var>
<op>	::=	+ | - | * | /
<coef>	::=	c1 | c2
<var>	::=	v1 | v2

Example: **{0, 1, 0, 0, 1, 1, 1, 1}**

Table S8: Example of translation.

Step	Codon	Symbol	Rule	Result
0			starting:	<expr>
1	0	<expr>	(<expr>)<op>(<expr>)	(<expr>)<op>(<expr>)
2	1	<expr>	<coef>*<var>	(<coef>*<var>)<op>(<expr>)
3	0	<coef>	c1	(c1*<var>)<op>(<expr>)
4	0	<var>	v1	(c1*v1)<op>(<expr>)
5	1	<op>	-	(c1*v1)-(<expr>)
6	1	<expr>	<coef>*<var>	(c1*v1)-(<coef>*<var>)
7	1	<coef>	c2	(c1*v1)-(c2*<var>)
8	1	<var>	v2	**(c1*v1)-(c2*v2)**

The analytic representation of non-linear dependence of a agronomic traits on climatic factors is build using N functions from "words" of length L according to:

- allowed words: predictor's name or operation on expressions on predictors,
- allowed operations: '+', '-', '*', '/',
- allowed expressions: X, (X - Const), 1/(X - Const),
- a linear combination of N functions is further build using LASSO.

$$\text{Model} \equiv \{< \text{predictor indices} >, < \text{coefficients} >\}_{N\cdot(L+1)}$$

S3 Simulations of basic model for groups of plants collected at different locations

S3.1 Adiyaman region

S3.1.1 Oyali sampling site

```
F_ttf{Oyali} = 9.20415+31.6154*dl_mean_sowing_10x
+15.2601* (dl_mean_sowing_5x5 - 1)
-48.3563*dl_max_sowing_x25x30+6.20454* (dl_min_sowing_x25x30 - 2)
+2.67915* u_mean_sowing_x5x15/(dl_mean_sowing_x10 - 1)
+40.2098* (1/(rrr_mean_sowing_x5x10 - 1) + 3)
-6.78333* (rrr_mean_sowing_x10x15 - 1)
```

S3.2 Diyarbakir region

S3.2.1 Cermik collection site

```
F_ttf{Cermik} = 7.72062-6.11519* (t_max_sowing_x10x15 - 1)
-13.6836* (dl_max_sowing_5x5 + 1) -1.40979* (t_sowing - 1)
-6.34709*rrr_mean_sowing_10x+2.73817*dl_mean_sowing_x10
+7.61564* u_sowing/(rrr_mean_sowing_x10 - 1) +20.9414*t_mean_sowing_x10
+8.6968* (1/(rrr_mean_sowing_x25x30 - 3e-06) + 1)
+7.69392* (1/(t_max_sowing_x5 - 245.975) + 1)
-4.22051* 1/(dl_min_sowing_5x5 - 12.9072) +7.78682* (t_mean_sowing_x25x30 - 5.99776)
+2.96977* (dl_mean_sowing_x10 - 0.0333693)
```

Figure S2: Distribution of time to flowering for the collection sites. For each collection site a curve connects points with y coordinate equal the number of samples for which the time to flowering falls into the interval centered at the x coordinate and with the range equal $15.7 = (221 - 64)/10$, where 221 and 64 are maximum and minimum of time to flowering period and 10 is a number of bins.

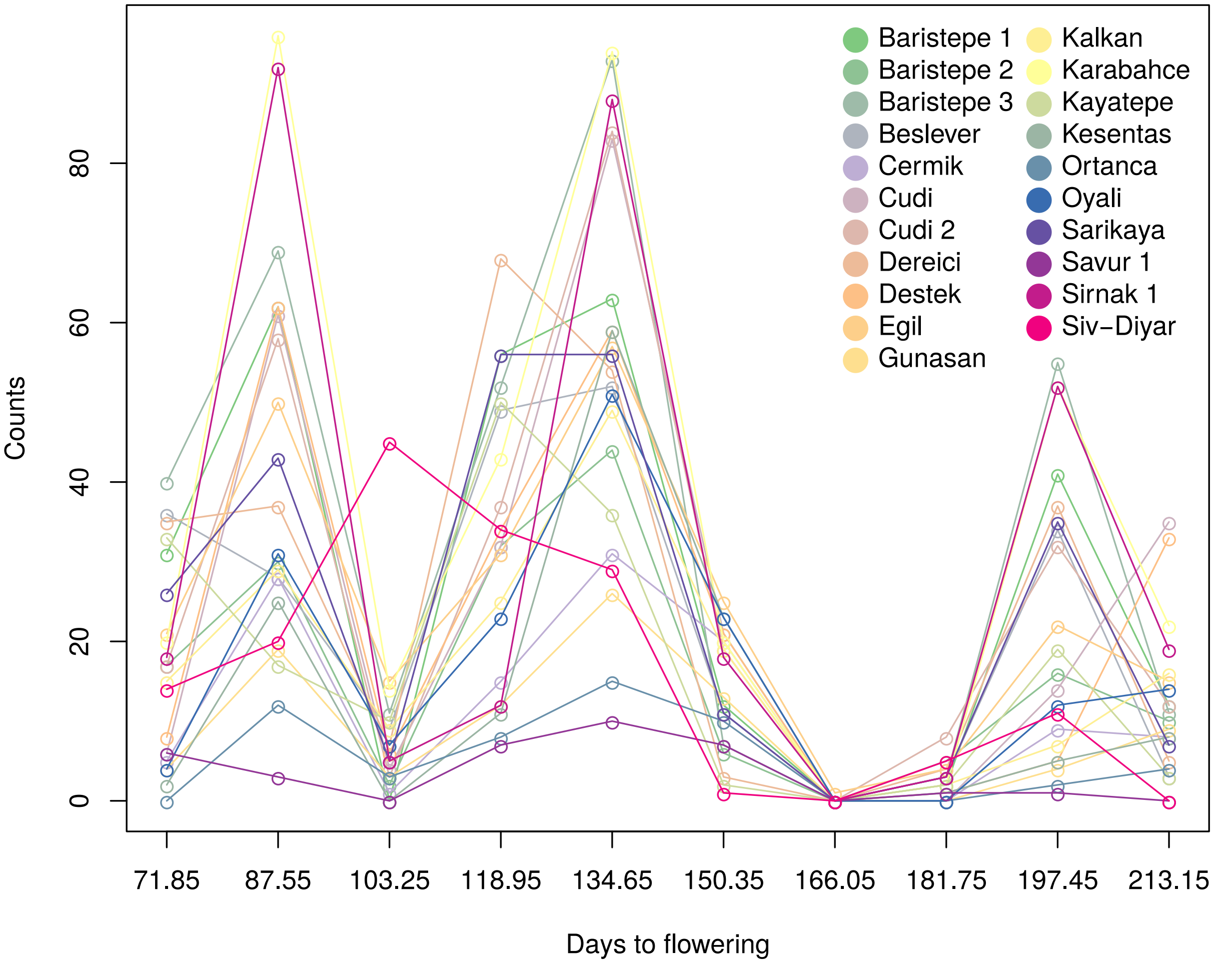

S3.2.2 Egil collection site

```
F_ttf{Egil}= -5.794269* dl_sowing+17.3173* (dl_max_sowing_5x5 - 1)
+3.02332*t_mean_sowing_x10x15+0.908939*rrr_mean_sowing_10x
+18.0502* (1/(t_mean_sowing_x15x20 - 15.4296) + 1) -12.3312* (rrr_mean_sowing_x10 - 2)
+11.855*t_max_sowing_10x-10.6967*t_max_sowing_x20x25
```

S3.2.3 Gunasan collection site

```
F_ttf{Gunasan}= -5.98337+1.49501* (u_sowing - 24.6368)
-14.0807*t_mean_sowing_x5x15-0.0853003* (rrr_mean_sowing_x15x20 + 1)
-0.573785* 1/(rrr_mean_sowing_x25x30 - 3e-06) +22.1211* (t_mean_sowing_x5x10 - 1)
-11.7507*rrr_mean_sowing_x5x10-1.38734* (1/(t_min_sowing_x5x10 - 1) + 1)
+0.838059* t_max_sowing_10x +2.69746* (dl_min_sowing_5x5 + dl_mean_sowing_x20x25 - 1)
+1.514* (t_max_sowing_5x5 + 1)
```

S3.2.4 Kalkan collection site

```
F_ttf{Kalkan}= 24.1499+60.3546* (dl_min_sowing_x25x30 - 1)
+132.999* (dl_mean_sowing_10x - 1) -114.092*dl_mean_sowing_x5
+138.089* (dl_max_sowing_x25x30 - 6.91648) -228.733* dl_min_sowing_x25x30
-4.78645* (rrr_mean_sowing_x5 + 1/(t_min_sowing_x10x15 - 1))
+358.952* (1/(t_mean_sowing_x15x20 - 28.1376) + 2)
```

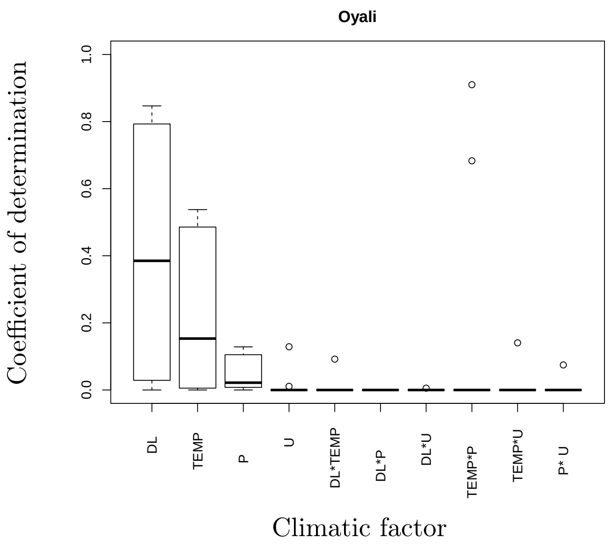

Figure S3: **Coefficient of determination of models, from which the terms that do not contain as a predictor the climatic factor or combination of factors analyzed were excluded.** The models were constructed for Oyali collection site. “DL”, “TEMP”, “P”, “U”, “DL*TEMP”, “DL*P”, “DL*U”, “TEMP*P”, “TEMP*U”, “P*U” correspond to factors related to day length, temperature, precipitation, humidity and their combinations, respectively.

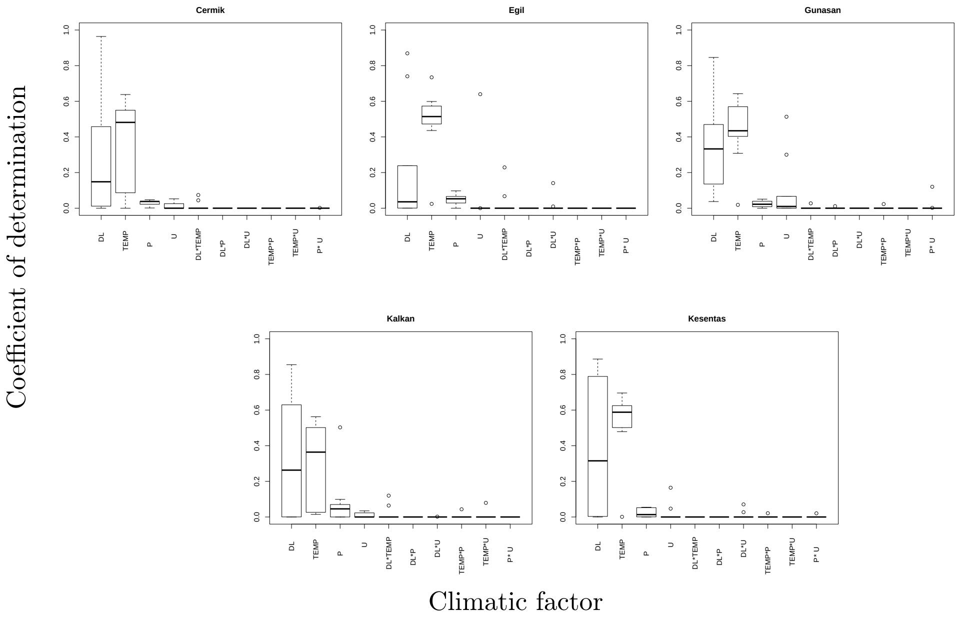

Figure S4: **Coefficient of determination of models, from which the terms that do not contain as a predictor the climatic factor or combination of factors analyzed were excluded.** Models were constructed for Diyarbakir region containing 5 collection sites: Cermik, Egil, Gunasan, Kalkan and Kesentas.“DL”, “TEMP”, “P”, “U”, “DL*TEMP”, “DL*P”, “DL*U”, “TEMP*P”, “TEMP*U”, “P*U” correspond to factors related to day length, temperature, precipitation, humidity and their combinations, respectively.

```
+357.969* (1/(t_min_sowing_x5x15 - 16.8238) + 2)
```

S3.2.5 Kesentas collection site

```
F= 2.67403*dl_min_sowing_x15x20
+6.30989* ( (1/(rrr_mean_sowing_10x - 5.27389))  +  (0 + 1) )
-0.395526* (dl_min_sowing_5x5 -  ( (1/(0 - 1))  - 1) )
-4.45295* ( ( (t_mean_sowing_x20x25 - 8.09734)  + 1)  * 1)
```

+1.97887* (rrr_mean_sowing_10x + ((0 - 1) * 1))
+0.179159* ((rrr_sowing * (0 + 1)) - 1)
+11.0441* ((((t_mean_sowing_x5 - 1) + 1) + 1) + 1) -8.19262*rrr_mean_sowing_5x5

S3.3 Mardin region

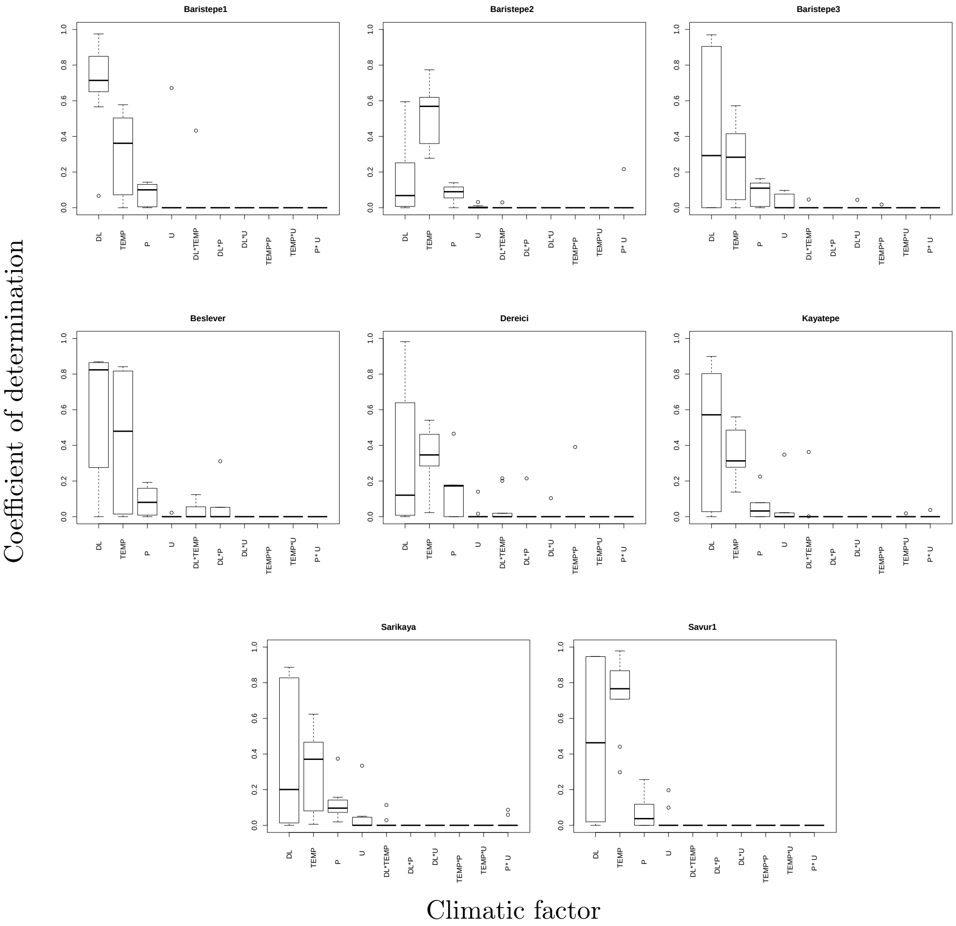

Figure S5: **Coefficient of determination of models, from which the terms that do not contain as a predictor the climatic factor or combination of factors analyzed were excluded.** Models were constructed for Mardin region containing 8 collection sites: Baristepe1, 2,3; Beslever, Dereici, Kayatepe, Sarikaya, Savur. "DL", "TEMP", "P", "U", "DL*TEMP", "DL*P", "DL*U", "TEMP*P", "TEMP*U", "P*U" correspond to factors related to day length, temperature, precipitation, humidity and their combinations, respectively.

S3.3.1 Baristepe1 collection site

F_ttf{Baristepe 1} = 13.8079* (t_mean_sowing_x5x10 + 2)
-6.2632* (rrr_mean_sowing_x20x25 - 1)
+1.77279* (1/(t_mean_sowing_x5 - 16.1607) + 1)
-6.10748*t_mean_sowing_x5x15
+9.51355* dl_min_sowing_x10
-7.06564*dl_min_sowing_x10
+13.9824* (1/(u_mean_sowing_x10 - 77.1469) + 1)

S3.3.2 Baristepe2 collection site

F_ttf{Baristepe 2} = 12.9608+36.2105* (dl_min_sowing_x10 - 1)
-27.4127* (dl_mean_sowing_5x5 - 1)
+14.2485* (rrr_sowing - 1)

```
+24.6977*t_mean_sowing_x5x10
-1.25134* (1/(t_max_sowing_x10 - 22.9178) + 1)
-19.5635*rrr_mean_sowing_x20x25
+11.9122* (dl_min_sowing_x10 - 0.768754)
-29.1344* (rrr_mean_sowing_x10x15 - 1)
-8.68211* (t_max_sowing_x20x25 - 3.49168) +1.03432*t_sowing
```

S3.3.3 Baristepe3 collection site

```
F_ttf{Baristepe 3} = 27.5014+28.7368* (1/(u_mean_sowing_x25x30 - 63.7807) + 1)
+31.504* (1/(t_mean_sowing_x15x20 - 1) + 2)
+15.0343* (1/(rrr_mean_sowing_x10x15 - 4.71447)) -1.19299* (t_sowing - 1)
+1.11872*dl_mean_sowing_x25x30+1.8825* (1/(t_max_sowing_10x - 3e-06))
+50.2752*dl_max_sowing_5x5-80.0478*dl_min_sowing_x15x20+27.7653*dl_max_sowing_5x5
```

S3.3.4 Beslever collection site

```
F_ttf{Beslever} = 4.30005-42.8547* (dl_mean_sowing_x25x30 - 6.84638)
-10.5078*rrr_mean_sowing_5x5+0.469497* (1/(t_min_sowing_x25x30 - 1))
+1.61085*t_mean_sowing_5x5+3.29597*dl_mean_sowing_10x
-0.965618* (t_min_sowing_x20x25 - 0.0184521)
+5.69087* (1/(t_min_sowing_x10 - 0.165043) + 1)
+29.2158* dl_max_sowing_x5
```

S3.3.5 Dereici collection site

```
F_ttf{Dereici} = 0.700877* (1/(dl_min_sowing_x10 - 9.53771) + 1)
-0.17589* (dl_sum_sowing_x15x20 + 1)
+0.193719* (u_mean_sowing_x25x30 + dl_sum_sowing_5x5)
+1.10745*t_mean_sowing_10x-0.162144* (dl_sum_sowing_x15x20 - 1)
+0.0377145* (dl_sum_sowing_10x - 127.874) +0.352585* (t_sowing + 2)
```

S3.3.6 Kayatepe collection site

```
F_ttf{Kayatepe}= -4.33184-73.9799* (dl_min_sowing_x25x30 - 5.79186)
+38.6528* (dl_mean_sowing_5x5 - 1) +1.6611* (u_mean_sowing_x10 / t_sowing)
-5.2006*rrr_mean_sowing_x15x20-9.42505* 1/(t_max_sowing_10x - 10.9558)
+13.5194* (dl_max_sowing_10x + 1) +9.35564* (dl_max_sowing_x20x25 - 6.13241)
-5.34758*t_mean_sowing_x5x10
```

S3.3.7 Sarikaya collection site

```
F_ttf{Sarikaya}= 6.37123+0.585271* (1/(u_mean_sowing_x5x10 - 84.0226) + 2)
-15.2554*dl_max_sowing_x25x30+32.7371* (1/(rrr_mean_sowing_x5x15 - 4.75898) + 1)
-2.22952*t_mean_sowing_x20x25-3.12324*t_mean_sowing_10x+7.38126*t_mean_sowing_x10
+3.58892*t_mean_sowing_x5+14.3045* dl_sowing
```

S3.3.8 Savur collection site

```
F= 33.0583-49.5816* ( ( (dl_min_sowing_x35x40 / 1)  * 1)  / 1)
-4.79387*dl_mean_sowing_x30x35+6.05608* (1/(t_min_sowing_x5x10 - 1.06819))
+7.15576* ( ( (dl_min_sowing_x5x15 - 0.0862557)  * 1)  / 1)
+1.42139*dl_mean_sowing_5x5+65.5277* (dl_min_sowing_x10 +  ( (0 - 1)  * 1) )
+1.64192* (1/(t_mean_sowing_10x - 0.209093)) +3.61597*t_mean_sowing_5x5
-6.39895*t_mean_sowing_5x5-1.38637* ( (t_min_sowing_x20x25 +  (0 / 1) )  * 1)
```

S3.4 Sirnak region

S3.4.1 Cudi collection site

```
F_ttf{Cudi} = 53.7292+10.16* 1/(rrr_sowing - 3e-06)
+3.61843*dl_mean_sowing_x10-2.87732* (rrr_mean_sowing_10x - 2)
```

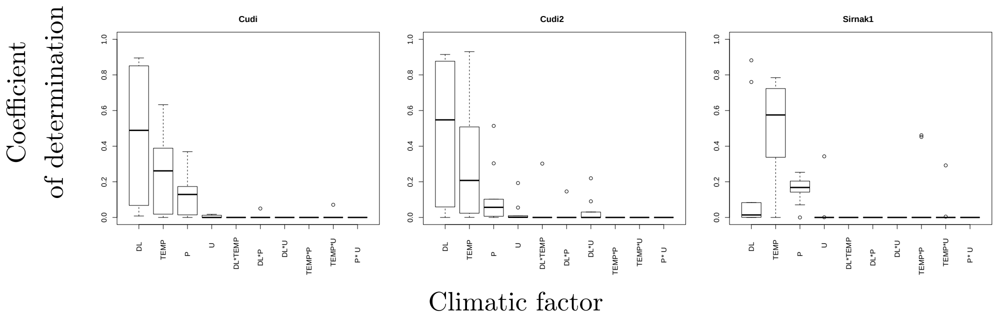

Figure S6: **Coefficient of determination of models, from which the terms that do not contain as a predictor the climatic factor or combination of factors analyzed were excluded.** Models were constructed for Sirnak region containing 3 collection sites: Cudi, Cudi2, Sirnak1. “DL”, “TEMP”, “P”, “U”, “DL*TEMP”, “DL*P”, “DL*U”, “TEMP*P”, “TEMP*U”, “P*U” correspond to factors related to day length, temperature, precipitation, humidity and their combinations, respectively.

```
+5.56862* 1/(t_max_sowing_10x - 11.6637) +61.1613* dl_min_sowing_x10
-65.0572* (dl_mean_sowing_x25x30 - 2) -9.21037* 1/(u_mean_sowing_x15x20 - 36.3213)
-2.23985*t_mean_sowing_x10x15
```

S3.4.2 Cudi2 collection site

```
F_ttf{Cudi 2} = 9.95629+11.6046* (1/(t_max_sowing_x10x15 - 3e-06) + 1)
-5.41255*t_min_sowing_x25x30+33.2861* 1/(rrr_mean_sowing_10x - 4.89121)
-3.3477* (t_max_sowing_x20x25 - 2)
+6.51924* (1/(t_min_sowing_5x5 - 1.58407) + 1)
+8.31581* dl_sowing -8.11504*dl_max_sowing_5x5
+12.44*t_mean_sowing_x10 +20.8716* 1/(rrr_mean_sowing_10x - 4.78037)
-2.37747* (1/(t_min_sowing_5x5 - 6.62519) + 1)
```

S3.4.3 Sirnak1 collection site

```
F= -3.93957*dl_mean_sowing_x25x30-4.94223*dl_min_sowing_x25x30
-7.64687* (rrr_mean_sowing_x10x15 *  (1/(rrr_mean_sowing_x20x25 - 1)) )
-2.74691* ( ( ( (t_mean_sowing_x10x15 - 1)  - 1)  * 1)  - 1)
+27.8742* ( (dl_mean_sowing_x10 +  (0 / 1) )  + 1)
-0.0310024* ( ( (1/(t_mean_sowing_5x5 - 7.45498))  + 1)  + 1)
-8.81769*rrr_mean_sowing_x25x30-52.6879* (dl_min_sowing_x20x25 - 4.43781)
+29.8705*dl_mean_sowing_5x5-0.359604* (1/(t_max_sowing_10x - 11.3058))
```

S3.5 Urfa region

S3.5.1 Destek collection site

```
F_ttf{Destek} = 60.5827* dl_mean_sowing_x5-78.1612* (dl_max_sowing_x20x25 - 1)
+2.59767*t_min_sowing_x25x30-6.56845* (1/(t_max_sowing_5x5 - 23.1649) + 1)
+12.8946* (dl_max_sowing_x10 - 1) +5.33821*dl_min_sowing_x15x20
+8.96668*dl_max_sowing_10x-3.67765* t_mean_sowing_x10x15
```

S3.5.2 Karabahce collection site

```
F_ttf{Karabahce}= 10.4139-4.10009*rrr_mean_sowing_5x5-63.7621* (dl_sowing - 1)
+4.16564* 1/(t_min_sowing_x10 - 9.64554)
+1.99503* (t_mean_sowing_x5x15 + 1/(t_mean_sowing_x25x30 - 1))
+12.4275* 1/(rrr_mean_sowing_x5 - 2.19114) -10.9505*dl_min_sowing_5x5
+142.147*dl_max_sowing_x10
-21.2247* 1/(u_mean_sowing_x15x20 - 36.2591) -6.19875* 1/(u_sowing - 29.0557)
+14.1252* 1/(t_mean_sowing_x15x20 - 5.14496) -80.1828* (dl_min_sowing_x25x30 - 3)
```

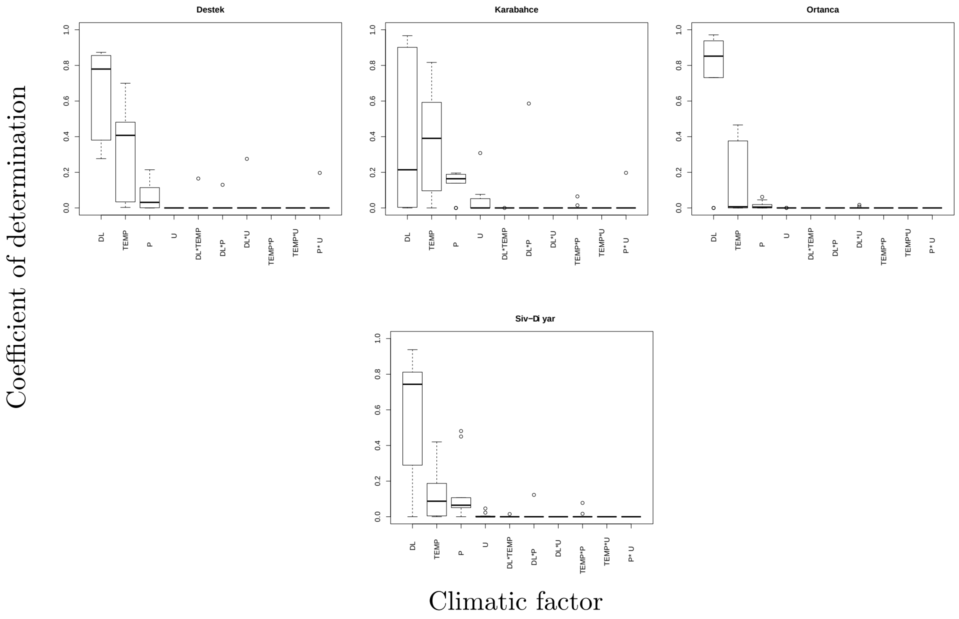

Figure S7: **Coefficient of determination of models, from which the terms that do not contain as a predictor the climatic factor or combination of factors analyzed were excluded.** Models were constructed for Urfa region containing 4 collection sites: Destek, Karabahce, Ortanca, Siv-Diyar. "DL", "TEMP", "P", "U", "DL*TEMP", "DL*P", "DL*U", "TEMP*P", "TEMP*U", "P*U" correspond to factors related to day length, temperature, precipitation, humidity and their combinations, respectively.

S3.5.3 Ortanca collection site

```
F_ttf{Ortanca}= -14.0091-17.0428* ( (dl_mean_sowing_x20x25 -  (0 * 1) )  * 1)
+4.40484*rrr_mean_sowing_x10+1.06432*rrr_mean_sowing_x25x30
-78.4099*dl_sowing+10.2068* (u_mean_sowing_x15x20 / dl_mean_sowing_x15x20)
-56.0995* (dl_mean_sowing_x25x30 +  (1/(dl_sum_sowing_x20x25 - 1)) )
-10.9819* (rrr_mean_sowing_x20x25 +  ( (0 + 1)  / 1) )
-14.0086* ( (1/(dl_sum_sowing_x5x10 - 294))  +  (0 + 1) )
+82.6341* (dl_max_sowing_x5x10 + dl_max_sowing_10x)
```

S3.5.4 Siv-Diyar collection site

```
F_ttf{Siv-Diyar}= 18.3292-54.4563* (dl_min_sowing_x25x30 - 3.16746)
-6.11188*rrr_mean_sowing_5x5+13.0569*dl_min_sowing_x5
-0.284159* (1/(dl_mean_sowing_5x5 - 9.35535))
-0.605242* (rrr_mean_sowing_x10 -  (1/(t_sowing - 1)) )
+3.21404* ( ( ( (1/(t_mean_sowing_x5x10 - 1))  + 1)  * 1)  - 1)
+19.2032* ( ( (1/(t_max_sowing_x10x15 - 8.57778))  - 1)  + 1)
+39.3192*dl_mean_sowing_x10
```

S4 Simulation of models for SNP groups

We build basic model for each group. Ten runs were performed with the same algorithmic parameters but different seeds for random number generator. In the following sections we present selected models that have the best coefficient of determination for each group.

Various agroclimatic factors and their combinations were selected as predictors by stochastic optimization. In order to be able to compare obtained models we cluster predictors into ten categories: "DL", "TEMP", "P", "U", "DL*TEMP", "DL*P", "DL*U", "TEMP*P", "TEMP*U", "P*U" – that correspond to factors related to day length, temperature, precipitation, humidity and there combinations, respectively.

We estimated the importance of the climatic factor category as the partial coefficient of determination calculated as the square of Pearson's correlation coefficient.

(a) **snp1G1** (b) **snp1G2**

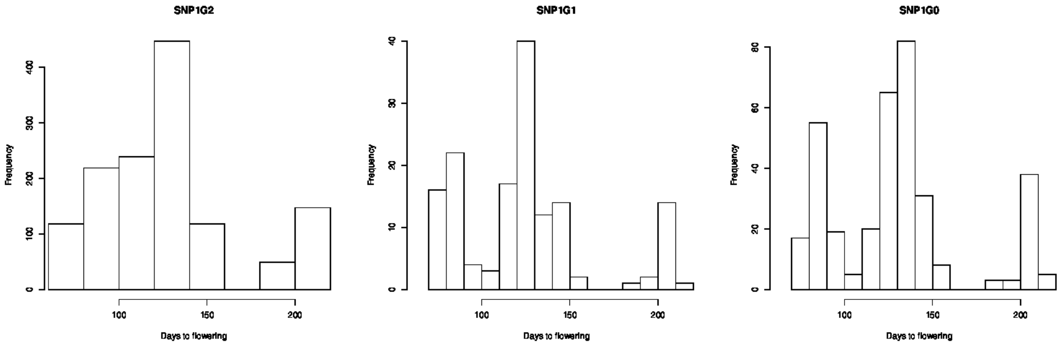

Figure S8: Distribution of time to flowering for the SNP1 group.

(a) **snp1G0**

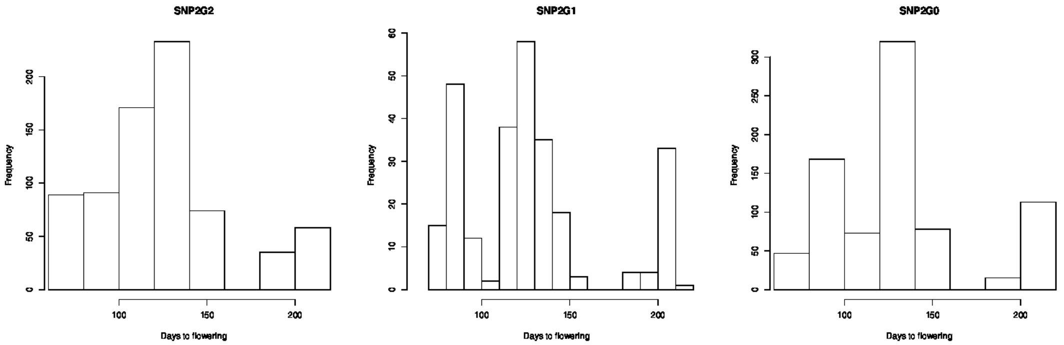

Figure S9: Distribution of time to flowering for the SNP2 group.

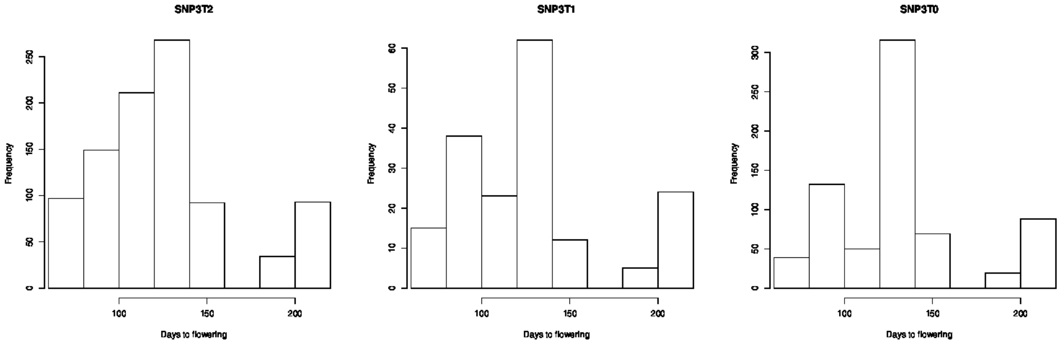

Figure S10: Distribution of time to flowering for the SNP3 group.

S4.1 SNP1 group

S4.1.1 REF/REF allele combination

```
F_ttf{1RR}= -1.73295+0.283651*u_mean_sowing_x10x15
-6.38755* (rrr_sowing + 1/(rrr_mean_sowing_x5x15 - 12.2178) + rrr_mean_sowing_x60 - 2)
+13.9961* (t_mean_sowing_x5x10 + dl_min_sowing_x5x15)
-0.515413*t_max_sowing_x5x10
-1.50788* (t_mean_sowing_x45 - 4.75863) * (dl_max_sowing_x5x10 - 1)
```

S4.1.2 REF/ALT allele combination

```
F_ttf{1RA}= -0.496638+1.83253* (dl_mean_sowing_x5 - 0.276982)
-1.7322*dl_min_sowing_x5x10+4.17823*t_mean_sowing_5x5
```

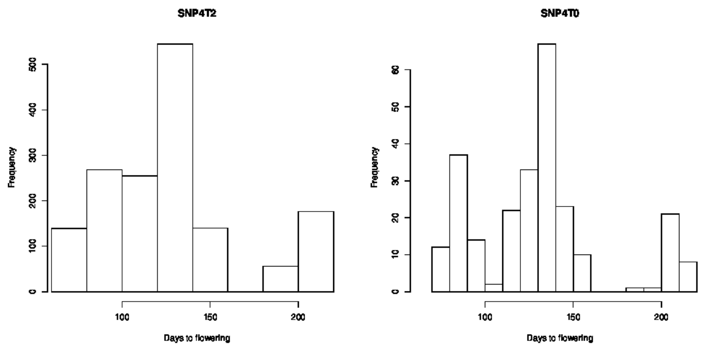

Figure S11: Distribution of time to flowering for the SNP4 group.

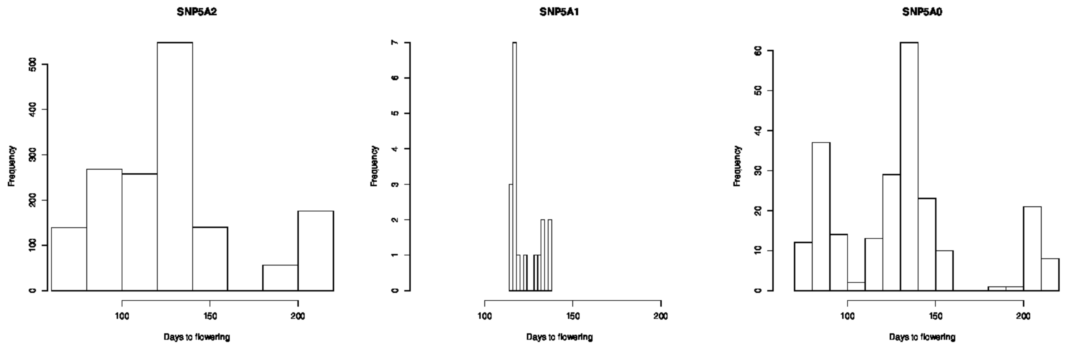

Figure S12: Distribution of time to flowering for the SNP5 group.

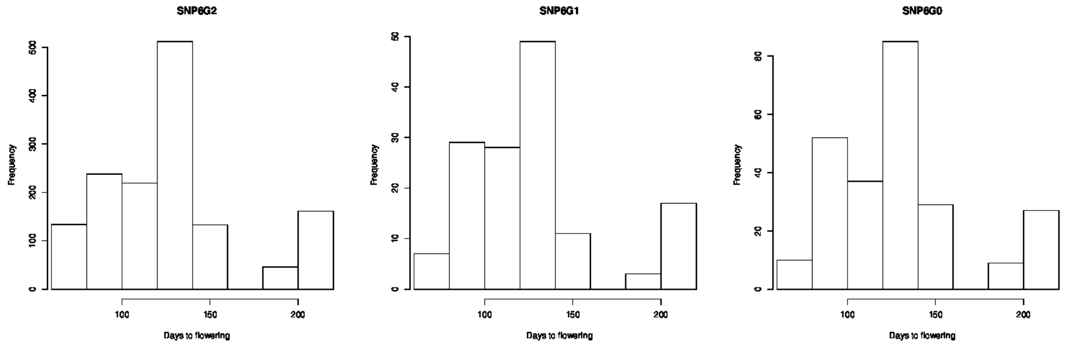

Figure S13: Distribution of time to flowering for the SNP6 group.

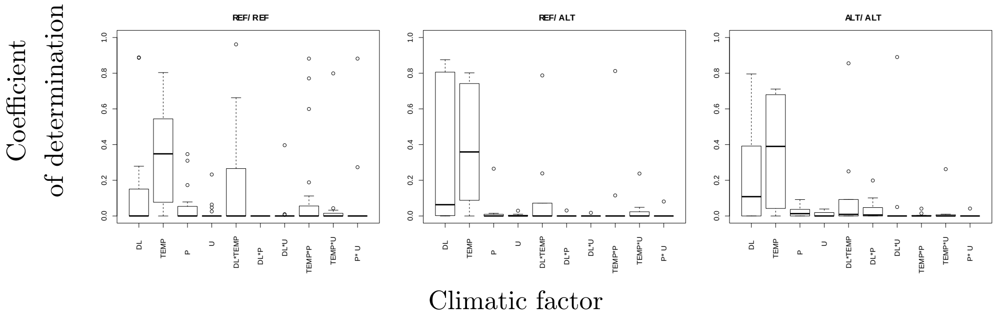

Figure S14: Coefficient of determination of models for the SNP1 group and from which the terms that do not contain as a predictor the climatic factor or combination of factors analyzed were excluded.“DL”, “TEMP”, “P”, “U”, “DL*TEMP”, “DL*P”, “DL*U”, “TEMP*P”, “TEMP*U”, “P*U” correspond to factors related to day length, temperature, precipitation, humidity and their combinations, respectively.

```
+0.387806* (u_mean_sowing_x5 - 34.0322) -5.48297* (1/(t_sowing - 12.5542) - 2)
+16.1149* (1/(dl_min_sowing_x45 - 9.33506) + 3)
```

S4.1.3 ALT/ALT allele combination

```
F_ttf{1AA}= -8.27235-14.398* (u_sowing / t_max_sowing_x25)
+13.6256*t_mean_sowing_x5x10
+0.878758* (dl_min_sowing_x15x20 + 1/(rrr_mean_sowing_x15x20 - 8.3997) - 1)
-14.0159*rrr_mean_sowing_x45+1.13909* (dl_min_sowing_x15x20 - 3e-06)
+12.8711* (u_mean_sowing_x5x10 / t_mean_sowing_x40 + 2)
```

S4.2 SNP2 group

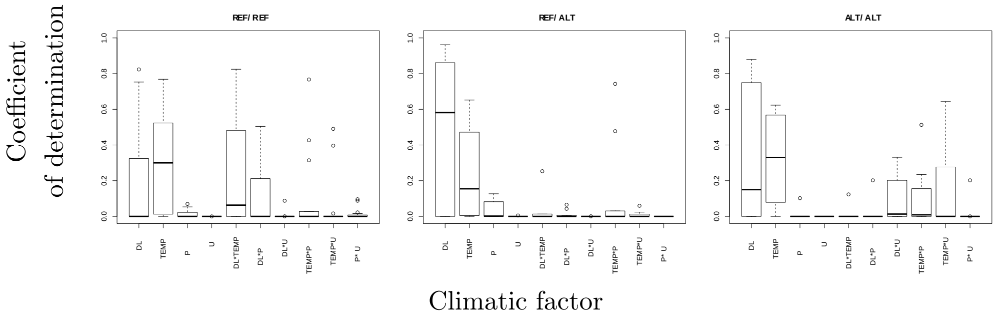

Figure S15: Coefficient of determination of models, for the SNP2 group and from which the terms that do not contain as a predictor the climatic factor or combination of factors analyzed were excluded. “DL”, “TEMP”, “P”, “U”, “DL*TEMP”, “DL*P”, “DL*U”, “TEMP*P”, “TEMP*U”, “P*U” correspond to factors related to day length, temperature, precipitation, humidity and their combinations, respectively.

S4.2.1 REF/REF allele combination

```
F_ttf{2RR}= 1.67929+0.934513*dl_min_sowing_x15x20-1.41556*t_mean_sowing_5x5
+13.5657* (1/(dl_min_sowing_x60 - 9.21829)) +6.72145*t_mean_sowing_x10
-4.68801* (rrr_mean_sowing_5x5 - rrr_sowing + 3)
+2.1578* (dl_mean_sowing_x60 + 1/(rrr_mean_sowing_x25x30 - 4.76846) - 1)
```

S4.2.2 REF/ALT allele combination

```
F_ttf{2RA}= 25.0841-12.7463*dl_max_sowing_x20x25-60.9916*dl_mean_sowing_x55
+43.7957* (1/(t_min_sowing_x35 - 4.61344) + 4)
-7.24295*rrr_mean_sowing_x60
+68.0418* (dl_mean_sowing_x5x10 - 1/(rrr_mean_sowing_x15x20 - 2.51888))
+31.642* (1/(t_mean_sowing_x20 - 3.25e-06) - 1/(u_mean_sowing_5x5 - 37.2475) + 1)
```

S4.2.3 ALT/ALT allele combination

```
F_ttf{2AA}= 6.33906-5.9889* (t_min_sowing_x5 + 2)
+22.0297*t_mean_sowing_x10-0.415907*t_mean_sowing_x10-2.22058*t_max_sowing_x10x15
-13.41669*(rrr_sowing - 0.00868082)
+1.06218*(1/(t_max_sowing_x5 - 20.8651) + u_mean_sowing_x25 - 4)
```

S4.3 SNP3 group

S4.3.1 REF/REF allele combination

```
F_ttf{3RR}= 0.836227+0.474944* (u_mean_sowing_x10 - t_min_sowing_x10 + 4.5444)
-5.2175* (1/(t_max_sowing_x15x20 - 21.2039) + 4)
+18.8528*u_mean_sowing_x55+6.63201*t_mean_sowing_x15x20
+0.245707* (1/(t_mean_sowing_x5 - 16.1581) + 4) -18.1631*u_mean_sowing_x35
```

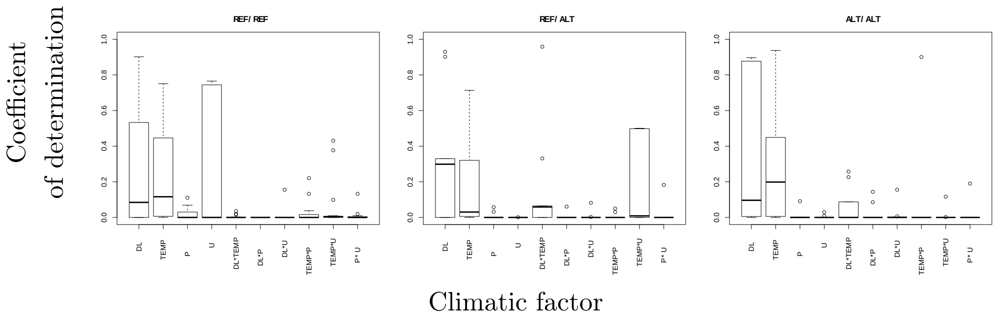

Figure S16: Coefficient of determination of models for SNP3 group and from which the terms that do not contain as a predictor the climatic factor or combination of factors analyzed were excluded. “DL”, “TEMP”, “P”, “U”, “DL*TEMP”, “DL*P”, “DL*U”, “TEMP*P”, “TEMP*U”, “P*U” correspond to factors related to day length, temperature, precipitation, humidity and their combinations, respectively.

S4.3.2 REF/ALT allele combination

```
F_ttf{3RA}= -38.016*dl_max_sowing_x20x25-34.2875*dl_mean_sowing_x55
-16.0546* (1/(dl_max_sowing_x55 - 17.9478) - 1/(t_max_sowing_5x5 - 34.1199) + 3)
-10.9494* (t_mean_sowing_x55 - 1) * (u_sowing - 164.47)/(u_sowing - 236.505)
+97.8709* (dl_mean_sowing_x5x10 - 1/(t_sowing - 2.51888))
-23.0135* (1/(dl_max_sowing_x15 - 3e-06) - 1/(t_max_sowing_x60 - 31.9392))
```

S4.3.3 ALT/ALT allele combination

```
F_ttf{3AA}= 23.4994-5.19775* (t_min_sowing_x20x25 - 3e-06)
+17.1881* (1/(u_sowing - 46.6227) + 6)
-22.9827* (1/(t_min_sowing_5x5 - 3e-06) + 6)
+10.3181* (t_sowing - 3e-06 + 1/(t_min_sowing_x25x30 - 0.783092) + 3)
```

S4.4 SNP4 group

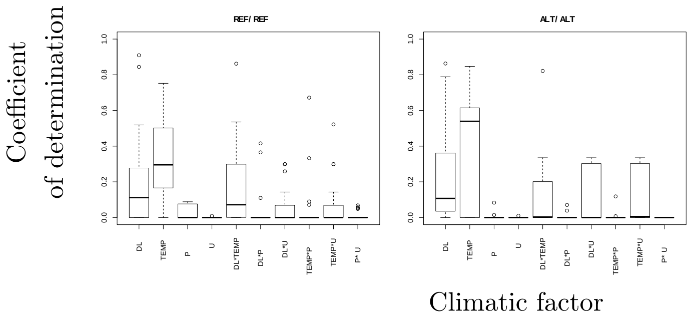

Figure S17: Coefficient of determination of models for SNP4 group and from which the terms that do not contain as a predictor the climatic factor or combination of factors analyzed were excluded. “DL”, “TEMP”, “P”, “U”, “DL*TEMP”, “DL*P”, “DL*U”, “TEMP*P”, “TEMP*U”, “P*U” correspond to factors related to day length, temperature, precipitation, humidity and their combinations, respectively.

S4.4.1 REF/REF allele combination

```
F_ttf{4RR}= 4.67892-4.09428* (t_sowing + 1/(t_sowing - 15.8739))
-0.0605318* (t_min_sowing_5x5 + 5)
+15.4048* (t_mean_sowing_5x5 + 5)
-22.1678* (dl_min_sowing_x15x20 - 15.4123 + rrr_mean_sowing_x35) -2.4386*t_sowing
```

S4.4.2 ALT/ALT allele combination

```
F_ttf{4AA}= -7.24322+0.063778* 1/(dl_max_sowing_x5x10 - 9.66774)
```

```
+1.94307* (t_mean_sowing_x10 - 4) +3.5069* (t_max_sowing_x5x10 + 2)
-1.69592* (1/(t_max_sowing_5x5 - 269.159) + dl_mean_sowing_x20 - 1)
+5.04824* (1/(dl_mean_sowing_x30 - 10.3235) + t_mean_sowing_x10 + 1)
```

S4.5 SNP5 group

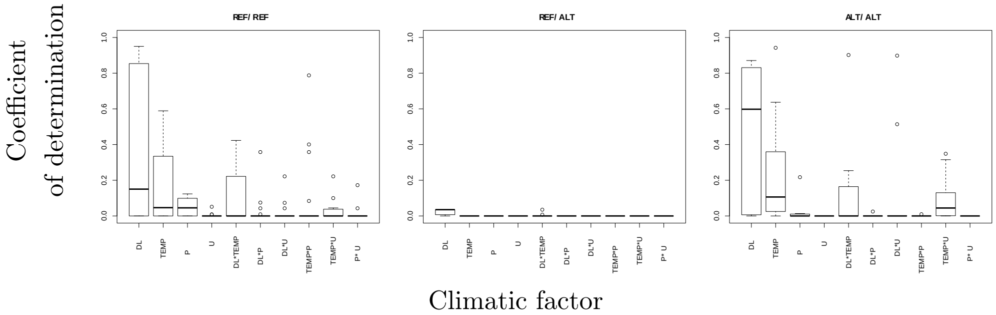

Figure S18: Coefficient of determination of models for the SNP5 group and from which the terms that do not contain as a predictor the climatic factor or combination of factors analyzed were excluded. “DL”, “TEMP”, “P”, “U”, “DL*TEMP”, “DL*P”, “DL*U”, “TEMP*P”, “TEMP*U”, “P*U” correspond to factors related to day length, temperature, precipitation, humidity and their combinations, respectively.

S4.5.1 REF/REF allele combination

```
F_ttf{5RR}= 10.4351+221.946* (0.5/(t_max_sowing_x10x15 - 30.6521)  + 1)
-94.7756* (2 * (dl_min_sowing_x15x20 - dl_mean_sowing_x5x15)  + 1)
+38.1847* (1/(rrr_mean_sowing_x35 - 3.89675))
-32.5479* ((u_mean_sowing_x15x20 - 45.7154)/(t_sowing - 65.4509) + 1)
```

S4.5.2 REF/ALT allele combination

```
F_ttf{5RA}= 0.118333*((t_min_sowing_x15x20 - 32.8199)*(dl_min_sowing_x45 - 12.1148)+
+dl_sum_sowing_x5)
```

S4.5.3 ALT/ALT allele combination

```
F_ttf{5AA}= -8.10567+57.5219* (dl_sowing - dl_min_sowing_x60 - 1)
-1.17285*t_mean_sowing_x15x20
+3.953675* (t_max_sowing_x10 - 5.5852)/(dl_max_sowing_x40 - 5.64865)
-2.78136* (t_max_sowing_x45 + 1/(t_mean_sowing_x10x15 - 13.7744) - 3)
-0.6* (t_max_sowing_x50 / u_mean_sowing_5x5)  /  (1/(t_max_sowing_x5x15 - 1))
```

S4.6 SNP6 group

S4.6.1 REF/REF allele combination

```
F_ttf{6RR}= 4.95377+12.5834*t_mean_sowing_x5x10-13.4134*rrr_mean_sowing_x20
-0.494951* (1/(t_max_sowing_x10x15 - 9.45389))
+33.6342* 1/(t_sowing - 18.4246)
-10.3318*t_mean_sowing_x55+0.340232*t_mean_sowing_x15x20
```

S4.6.2 REF/ALT allele combination

```
F_ttf{6RA}= -1.69818-33.9587*dl_min_sowing_x20x25
-20.7909* (dl_max_sowing_x10/(dl_sum_sowing_x25x30 - 292.05) + dl_min_sowing_x40)
-1.98932* (t_max_sowing_x55 - 15.6494)
+62.5304* (dl_mean_sowing_5x5 + 1/(t_sowing - 19.1224) + 1)
```

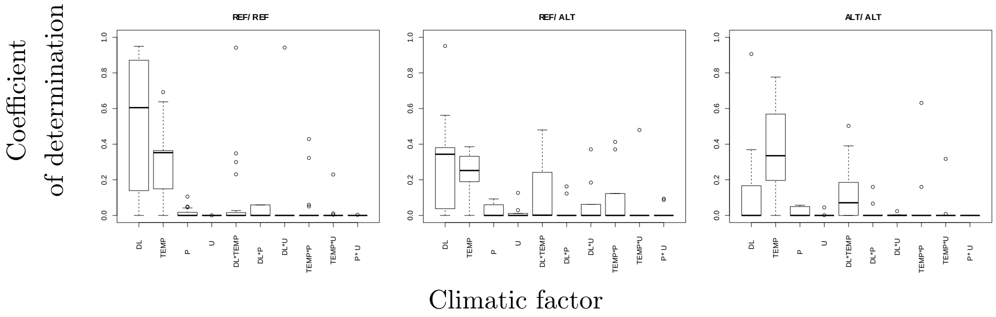

Figure S19: Coefficient of determination of models for SNP6 group and from which the terms that do not contain as a predictor the climatic factor or combination of factors analyzed were excluded. “DL”, “TEMP”, “P”, “U”, “DL*TEMP”, “DL*P”, “DL*U”, “TEMP*P”, “TEMP*U”, “P*U” correspond to factors related to day length, temperature, precipitation, humidity and their combinations, respectively.

S4.6.3 ALT/ALT allele combination

```
F_ttf{6AA}= -5.22884*dl_max_sowing_x55
-0.514132* (t_min_sowing_x10x15 + 1/(u_mean_sowing_x5x15 - 72.0772) +
 + (t_max_sowing_x10x15 - 8.67654))
+0.818053* (dl_max_sowing_5x5 - t_mean_sowing_5x5 + u_mean_sowing_x45 - 2)
+6.09927* dl_max_sowing_x40 *  (dl_max_sowing_5x5 - dl_min_sowing_x20x25)
```

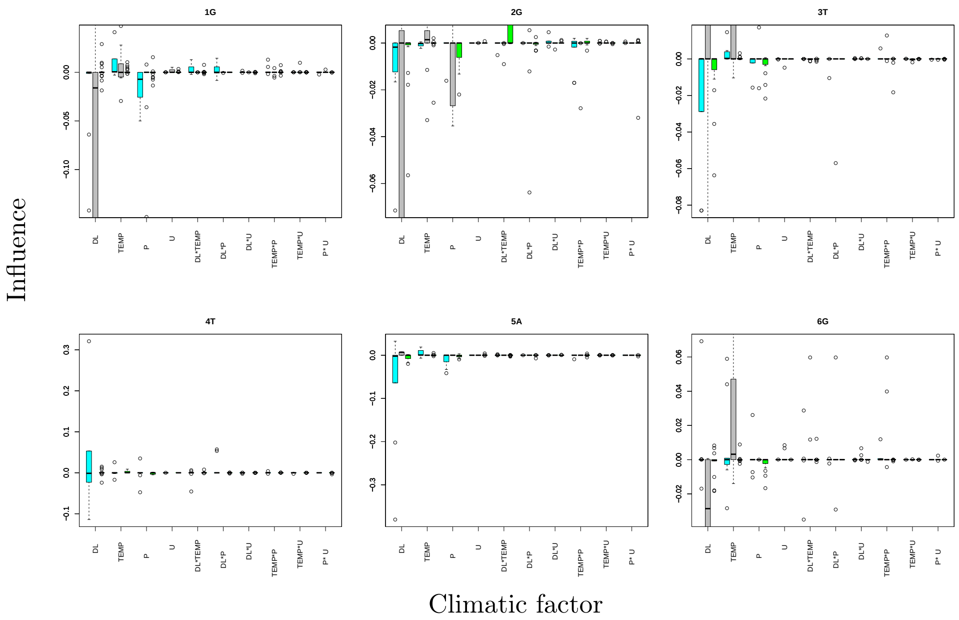

Figure S20: Factor influence estimators calculated for model ensembles. Each factor influence on phenotype was estimated as a finite difference approximation of the partial derivative of a regression function in respect to the factor. Both the direction and extent are important. Colors cyan, gray and green are used for **ALT/ALT**, **REF/ALT** and **REF/REF** allele cimbinations respectively.

S5 Differential Evolution Entirely Parallel method

An effective stochastic method for function minimization termed Differential Evolution (DE), proposed in [Storn and Price, 1995], operates on a set (population) parameter vectors (individuals). The initial population is generated randomly, a size of population NP is fixed. DEEP one can be applied to solve both unconstrained and constrained optimization problems. Constraints may be imposed in the form of inequalities or equalities for a subset of parameters or their combinations.

DEEP method [Kozlov and Samsonov, 2011] incorporates the “trigonometric mutation” rule proposed in [Fan and Lampinen, 2003] and used to take into account a value of the objective function for each

individual at the recombination step, and the adaptive scheme for selection of internal parameters based on the control of the population diversity developed in [Zaharie, 2002].

In DEEP the age of individual is defined as a number of iterations, during which the individual survived without changes. The number of oldest individuals is substituted with the same number of the best ones after the predefined number of iterations to avoid local minima. Calculations are terminated when the objective function variation becomes less than a predefined value during the several consecutive steps or the maximal number of iterations is exceeded.

DE operates on floating point parameters, while two algorithms for parameter conversion from real to integer are implemented in DEEP [Kozlov et al., 2013]. The first method rounds off a real value to the nearest integer number. Another two step procedure firstly sorts parameters in ascending order and then uses the indices as integer parameters.

DEEP employs the pool of worker threads with asynchronous queue of tasks to evaluate the individual solutions in parallel. The calculation of objective function for each trial vector is pushed to the asynchronous queue and starts as soon as there is an available thread in the pool [Kozlov et al., 2016].

DEEP is implemented in C programming language as console application and using interfaces from GLIB project `https://developer.gnome.org/glib/`, e.g. *Thread Pool API*. DEEP method is an open source and free software distributed under the terms of GPL licence version 3. The sources are available at `https://gitlab.com/mackoel/deepmethod`.

References

[Aho et al., 2006] Aho, A. V., Lam, M. S., Sethi, R., and Ullman, J. D. (2006). *Compilers: Principles, Techniques, and Tools (2Nd Edition)*. Addison-Wesley Longman Publishing Co., Inc., Boston, MA, USA.

[Fan and Lampinen, 2003] Fan, H.-Y. and Lampinen, J. (2003). A trigonometric mutation operation to differential evolution. *Journal of Global Optimization*, 27:105–129.

[Kozlov et al., 2013] Kozlov, K., Ivanisenko, N., Ivanisenko, V., Kolchanov, N., Samsonova, M., and Samsonov, A. M. (2013). Enhanced Differential Evolution Entirely Parallel Method for Biomedical Applications. *LNCS 7979*, V. Malyshkin (Ed.): PaCT 2013:409–416.

[Kozlov and Samsonov, 2011] Kozlov, K. and Samsonov, A. (2011). DEEP – Differential Evolution Entirely Parallel Method for Gene Regulatory Networks. *Journal of Supercomputing*, 57:172–178.

[Kozlov et al., 2016] Kozlov, K., Samsonov, A. M., and Samsonova, M. (2016). A software for parameter optimization with differential evolution entirely parallel method. *PeerJ Computer Science*, 2:e74.

[Noorian et al., 2016] Noorian, F., de Silva, A., and Leong, P. (2016). gramEvol: Grammatical Evolution in R. *Journal of Statistical Software, Articles*, 71(1):1–26.

[O'Neill and Ryan, 2001] O'Neill, M. and Ryan, C. (2001). Grammatical evolution. *IEEE Transactions on Evolutionary Computation*, 5(4):349–358.

[Storn and Price, 1995] Storn, R. and Price, K. (1995). Differential evolution – a simple and efficient heuristic for global optimization over continuous spaces. Technical Report Technical Report TR-95-012, ICSI.

[Zaharie, 2002] Zaharie, D. (2002). Parameter adaptation in differential evolution by controlling the population diversity. In Petcu, D., editor, *Proc. of 4th InternationalWorkshop on Symbolic and Numeric Algorithms for Scientific Computing*, pages 385–397, Timisoara, Romania.
